# The DDX6–4E-T interaction mediates translational repression and P-body assembly

**DOI:** 10.1093/nar/gkw565

**Published:** 2016-06-24

**Authors:** Anastasiia Kamenska, Clare Simpson, Caroline Vindry, Helen Broomhead, Marianne Bénard, Michèle Ernoult-Lange, Benjamin P. Lee, Lorna W. Harries, Dominique Weil, Nancy Standart

**Affiliations:** 1 Department of Biochemistry, University of Cambridge, Tennis Court Road, Cambridge CB21QW, UK; 2 Sorbonne Universités, UPMC, CNRS, IBPS, Developmental Biology Laboratory, 75005 Paris, France; 3 Institute of Biomedical and Clinical Sciences, University of Exeter Medical School, Barrack Road, Exeter EX2 5DW

## Abstract

4E-Transporter binds eIF4E via its consensus sequence YXXXXLΦ, shared with eIF4G, and is a nucleocytoplasmic shuttling protein found enriched in P-(rocessing) bodies. 4E-T inhibits general protein synthesis by reducing available eIF4E levels. Recently, we showed that 4E-T bound to mRNA however represses its translation in an eIF4E-independent manner, and contributes to silencing of mRNAs targeted by miRNAs. Here, we address further the mechanism of translational repression by 4E-T by first identifying and delineating the interacting sites of its major partners by mass spectrometry and western blotting, including DDX6, UNR, unrip, PAT1B, LSM14A and CNOT4. Furthermore, we document novel binding between 4E-T partners including UNR-CNOT4 and unrip-LSM14A, altogether suggesting 4E-T nucleates a complex network of RNA-binding protein interactions. In functional assays, we demonstrate that joint deletion of two short conserved motifs that bind UNR and DDX6 relieves repression of 4E-T-bound mRNA, in part reliant on the 4E-T-DDX6-CNOT1 axis. We also show that the DDX6-4E-T interaction mediates miRNA-dependent translational repression and *de novo* P-body assembly, implying that translational repression and formation of new P-bodies are coupled processes. Altogether these findings considerably extend our understanding of the role of 4E-T in gene regulation, important in development and neurogenesis.

## INTRODUCTION

4E-Transporter (EIF4ENIF1) is a large conserved metazoan protein, which first came into prominence as a factor that binds eIF4E, the translation initiation factor with high affinity for the m^7^G cap structure of eukaryotic mRNAs (1). During translation initiation, eIF4E interacts with eIF4G, a large scaffold protein that also binds the eIF4A RNA helicase, and eIF3 associated with the 40S ribosomal subunit. This interaction network links the 5′ end of mRNA with the small ribosomal subunit and, with the unwinding activity provided by eIF4A, enables scanning of the 5′ untranslated region (UTR) and recognition of the initiator AUG (2). In addition to recruiting eIF4A to the 5′ cap, eIF4E has recently been shown to also stimulate its activity in a cap-independent manner (3). eIF4E levels, particularly important for translation of mRNAs with structured 5′ UTR, are typically limiting for initiation, but are deregulated in cancer, senescence and autism (4–6).

One of the significant ways this ribosome relay is regulated is by controlling the eIF4E-eIF4G interaction mediated by the consensus motif YX_4_Lϕ in eIF4G. This motif, present in additional eIF4E-binding proteins, including the small 4E-BP (eIF4E-binding) protein family and 4E-Transporter (4E-T), competitively prevents the productive binding of eIF4E to eIF4G, hence reducing protein synthesis (5). Recent studies extended our understanding of this competition by demonstrating that 4E-BPs, 4E-T and related proteins all possess a nearby eIF4E-binding site downstream of YX_4_Lϕ. Notably, this second, non-canonical site is absent from eIF4G, and allows 4E-BPs and 4E-T to efficiently displace eIF4E from eIF4F (7–9).

4E-T proteins and the related *Drosophila* protein Cup are also understood to control access of ribosomes to the 5′ cap of specific mRNAs by interacting with 3′ UTR-RNA-binding proteins (reviewed in (4,6)). For example, *Drosophila* Cup represses *oskar* and *nanos* mRNAs in oogenesis by bridging Bruno and Smaug respectively which recognize sequences within their 3′ UTRs and eIF4E, thus precluding eIF4G recruitment of the small ribosomal subunit (10–12). In *C. elegans*, the 4E-T homologue IFET-1 (Spn2) interacts with OMA1/2 RNA-binding proteins to inhibit *mei-1* and *zif-1* mRNAs in oocytes (13,14). Indeed, IFET-1 functions as a broad-based co-repressor throughout germline development, and promotes large germline RNP granule condensation (15). Finally, we previously showed that in *Xenopus* oocytes, 4E-T is a component of the large CPEB translation repressor RNP complex that also contains Xp54/DDX6 RNA helicase, PAT1 and LSM14 proteins (16).

Levels of 4E-T/Cup proteins are particularly high in germ cells and early development when translational control predominates in gene regulation (6). Mutations in *Eif4enif1*, likely resulting in nonsense-mediated decay of 4E-T mRNA, have recently been detected in a family with premature ovarian failure (17). Interestingly, in a large RNAi screen in mouse oocytes, 4E-T was found to be essential for the breakdown of the nuclear envelope and resumption of meiosis (18). Furthermore, murine 4E-T determines the genesis of neurons from precursors by translationally repressing a proneurogenic transcription program (19), as part of a complex including Smaug2 (20), reminiscent of *Drosophila* Cup-Smaug interactions (12).

In addition to down-regulating protein synthesis, mammalian 4E-T has been shown to enhance decay of specific mRNAs such as those bearing 3′ UTR AU-rich elements (ARE) or microRNA-binding sites, in an eIF4E-dependent manner (21–23).

The name given to 4E-T reflects its characterized nucleocytoplasmic shuttling activity, via identified NLS and NES sequences (1). At steady state, mammalian 4E-T is found enriched in cytoplasmic Processing Bodies (P-bodies), and its overexpression enhances eIF4E concentration therein (7,21,24–26). P-bodies are understood to participate in mRNA storage as well as decay, and contain many RNA-binding proteins including DDX6, PAT1 and LSM14, phosphohydrolases such as the Dcp1/2 decapping enzyme, the 5′-3′ exonuclease Xrn1, and the translation initiation factor eIF4E, with ribosomes and other translation factors being excluded from these granules (27,28). Recently, we have identified C-terminal sequences in human 4E-T that promote its localization in P-bodies and these are conserved in *Drosophila* and *C. elegans* 4E-T proteins, but not in *Drosophila* Cup (26).

We and others have investigated how 4E-T proteins regulate expression of mRNAs to which they are bound, in the tether function assay, mimicking their normal recruitment to the 3′ UTR. Human 4E-T repressed bound reporter mRNA translation, rather than resulting in its decay, and unexpectedly, did so even when mutated to prevent interaction with eIF4E (26). Similar results were obtained for tethered *Drosophila* Cup in S2 cells (29). On the other hand, overexpression of (untethered) 4E-T reduces protein synthesis globally, in an eIF4E-dependent manner (21,26,30). Thus 4E-T exerts both eIF4E-dependent and eIF4E-independent effects on translation. We also demonstrated that 4E-T participates in microRNA-mediated silencing and co-precipitates several subunits of the CCR4-NOT complex (26), a key downstream effector of miRISC (reviewed in (31)). Interestingly, CNOT1 binds to and stimulates the DDX6 RNA helicase ATPase activity, thus mediating microRNA-mediated silencing (31–34). Moreover, as DDX6 is a well-characterized and direct partner of 4E-T (16,35,36), this network of interactions may underlie miRISC repression of translation initiation.

Here, we extend our analysis of the repression mechanism mediated by 4E-T. Using a combination of co-immunoprecipitation and mass spectrometry experiments in HEK293 cells we first identify major 4E-T interacting proteins, including DDX6, UNR, LSM14A and PAT1B, and map their binding sites. We show in the tether function assay that deletion of both DDX6 and UNR binding sites in 4E-T prevents its repression activity. More generally, we show that the 4E-T-DDX6 interaction also mediates miRNA translational silencing and *de novo* P-body assembly. Altogether, our data suggest that translational repression, including that mediated by microRNA, and P-body formation are coupled processes.

## MATERIALS AND METHODS

### Plasmids

NHA-tagged GFP and human 4E-T plasmids, wild-type and ΔCHD, were described in (26). NHA-4E-T deletion constructs, missing motif I-III, singly and in combination with loss of CHD, were obtained using chimeric PCR and primers with complementary ends. The deletion boundaries (I (131–161), CHD (208–245), II (291–316) and III (331–346)), were chosen on the basis of conservation, predicted secondary structure, and where possible started and/or ended at nearby proline residues.

All FLAG-tagged plasmids were constructed with the N-terminal 3x-CMV(E7533) 7.1 vector (Sigma Aldrich). cDNAs encoding human 4E-T (26), UNR and unrip (37) and DDX6 (gift from Reinhard Luhrmann) were subcloned with appropriate oligos using standard PCR techniques into the NotI/BamHI sites of 3x-CMV. The 4E-T deletion constructs, missing motif I-III and the CHD, were obtained by standard PCR using the NHA-deletion plasmids as templates. The 4E-T truncated fragment constructs were obtained using the wild-type 4E-T as template, except in the case of 4E-T1-300 and 4E-T 1–440 when the template was the NLS mutant of 4E-T described in (26). FLAG-UNRΔC (UNR1-744) was obtained using standard PCR techniques.

Complementing siRNA-resistant HA-tagged DDX6 plasmids, wild type and mutants in helicase motifs required for ATP hydrolysis (catal1-4) and CNOT1 binding (mif3), were a gift from Witek Filipowicz, and described previously (33). RL-Hmga2, wt and mutant, and Caf1_catal_/Pan2_catal_ plasmids were also gifts of Witek Filipowicz, and described previously (33).

### HEK293 cell culture and transfection

Human embryonic kidney HEK293T cells were maintained in DMEM supplemented with 10% fetal calf serum. Knockdown of protein in HEK293T cells was carried out using a 2-hit siRNA transfection protocol. On day 1, cells were seeded in either 35 mm 6-well plates (tether function assay) or 10 cm plates (immunoprecipitation) at a density of 2×10^5^ cell/ml and immediately transfected with the siRNA. Transfection of siRNA was performed as described for DNA transfection (26), using Lipofectamine 2000, according to manufacturer's instructions. Typically, 3 or 15 μg of siRNA were used per sample in 35 mm and 10 cm plates respectively. On day 2, the medium was replaced and a total of 1 or 5 μg (for 35 mm and 10 cm plates respectively) of the appropriate plasmid DNA was mixed with the siRNA and transfection was repeated. Cells were harvested on day 4. The efficiency of knockdown by RNAi was assessed by Western blotting. The sequences of the siRNAs, 5′-3′, were as follows: unrip 1:1 mix of AAACUGUUACGCAUAUAUGAC[dT][dT] and AACUUAUGGACGAUCUAUUGC[dT][dT] (38); UNR Santa Cruz, sc-76809, DDX6 GGAACUAUGAAGACUUAAA[dT][dT] and β-globin GGUGAAUGUGGAAGAAGU[dT][dT (39).

### Mass spectrometry

HEK293T cells were plated onto 10 cm plates at 3 × 10^5^ cells/ml and Lipofectamine 2000-transfected with either FLAG-4E-T or empty vector plasmids 24 h after plating. After a further 24 h, cells were harvested and the tagged proteins purified with 80 μl anti-M2 affinity gel according to manufacturer's instructions (Sigma-Aldrich). Bound proteins were eluted using 200 μl of 300 μg/ml FLAG peptide (Sigma), and separated briefly by SDS-PAGE. Each lane was sliced into four to six slices, and tryptic peptides obtained by in-gel digestion were identified using GELC/MS/MS mass spectrometry in the Cambridge Centre for Proteomics. Peptide analysis was carried out with Thermo Scientific Protein Center release 3.9 and UniProtHuman_Jan13. GO analysis of approx. 200 genes (Supplementary Table S1) was performed with http://amigo.geneontology.org/rte.

### Immunoprecipitation and western blot analysis

Immunoprecipitation to assess binding of specific co-factors of -tagged proteins was performed with lysates from transfected cells grown in 10 cm plates. Following the manufacturer's recommended conditions, lysates prepared from HEK293T cells were incubated with anti-M2 beads (Sigma-Aldrich), and after washing, bound proteins were eluted with SDS loading buffer. When indicated, the transfected lysates were supplemented with RNAse A (Life Technologies) at 40 μg/ml final concentration during the immunoprecipitation binding step.

Western blotting analysis was performed with 15% SDS-PAGE. The following antibodies were used in ECL, rabbit anti-4E-T (Abcam), DDX6 (Bethyl labs), UNR (gift from Anne Willis; Novus), unrip (gift from Richard Jackson; (37)), LSM14A (Millipore), PAT1B, (40; Bethyl), CNOT4 (Abcam), XRN1 (gift from Jens Lykke Andersen), actin (Sigma); mouse anti-FLAG (Sigma), mouse anti-eIF4E1 (Santa Cruz); rat anti-HA (Roche), tubulin (Abcam), goat anti-4E-T (Abcam); chicken anti-eIF4E2 (7).

### Tether function assay

The 4E-T tether function assay was described in detail in (26). Briefly, HEK293 cells were typically co-transfected with NHA-plasmids (control GFP and 4E-T) and two luciferase reporter plasmids encoding *Renilla* luciferase-Box B and the control firefly luciferase mRNAs. After 36–48 h, lysates were prepared and luciferase activities determined using the Dual luciferase system. All reported experiments were repeated at least three times, and in the case of the shown experiments, the average and standard deviation of the *Renilla* to firefly luciferase ratio is of three technical replicates. Levels of reporter mRNA in the tether function assay were quantitated by qRT-PCR, as described previously (26). Samples were also analysed by western blotting, as above.

### miRNA reporter assay

For 4E-T knockdown and rescue experiments with miRNA reporters, and based on similar assays with DDX6 (33), 300 000 HeLa cells per well of a six-well plate were first transfected with 3 μg of siRNA using 3 μl of Lipofectamine 2000 (Thermo Fisher Scientific). The 4E-T siRNA was [5′-CAGUCGAGUGGAGUGUACAUUGUdTdT; Thermo Scientific], and the control β-globin siRNA is specified above. A second transfection was performed 48 h later, using 80 000 cells per well of a 24-well plate and 0.75 μl of Lipofectamine 2000. The second transfection mixtures contained 0.25 ng of pSF3 CMV-RL-Hmga2-wt or -mut; 20 ng of Firefly luciferase firefly luciferase, 150 ng of plasmids encoding PAN2catal and CAF1catal, 100 ng of plasmid encoding either HA-GFP, or HA-4E-T (wt or mutant forms; all siRNA-resistant), and 1 μg of either 4E-T-specific or control siRNA. Cells were lysed 48 h after the second transfection and luciferase activities determined by the Dual luciferase assay. The levels of GFP and 4E-T (wt and mutants) proteins were monitored by western blotting.

### HeLa cell culture and transfection for immunofluorescence

HeLa cells were first transfected at the time of their plating with 1.5 μg siRNA (MWG Biotech) per 35 mm diameter dish using Lullaby (OZ Biosciences, France), and split in three 15 h later. Twenty four hours after siRNA delivery, cells were transfected with 1 μg of plasmid DNA using Genjet (TEBU). Sixty four hours after plating, cells were fixed for immunofluorescence or harvested for protein preparation. The sequence of the siRNA targeting the 3′ UTR region of 4E-T mRNA was 5′-3′: UGGUCUUUCUUUUUUGUAAdTdT.

### Immunofluorescence

Cells grown on glass coverslips were fixed in methanol for 3 min at −20°C. After rehydration, cells were incubated with the primary antibody for 1 h, rinsed with PBS, incubated with the fluorochrome-conjugated secondary antibody for 1 hour, and rinsed with PBS, all steps being performed at room temperature. Slides were mounted in Citifluor (Citifluor, UK). Microscopy was performed on a Leica DMR microscope (Leica) using a 63 × 1.32 oil immersion objective. Photographs were taken using a Micromax CCD camera 13 (Princeton Instruments) driven by Metamorph software. Images were processed with ImageJ. To quantitate P-bodies, we used the plugin Spot Detector of the open bioimage informatics platform Icy (http://icy.bioimageanalysis.org; (41)). Primary antibodies were goat 4E-T and rabbit EDC3 (Abcam), rabbit DDX6 (Novus), mouse FLAG M2 (Sigma). Secondary antibodies were purchased from Jackson ImmunoResearch Laboratories.

### Protein extraction and western blot analysis

Cytoplasmic proteins were extracted from HeLa cells and analyzed by western blotting as described in (35). Primary antibodies included goat 4E-T (Abcam), mouse FLAG M2 (Sigma) and rabbit ribosomal S6 protein (Cell Signaling Technology). Secondary antibodies were purchased from Jackson ImmunoResearch Laboratories.

## RESULTS

### Proteomic analysis reveals the major interacting factors of 4E-T to be DDX6, UNR and unrip

**Figure 1.**
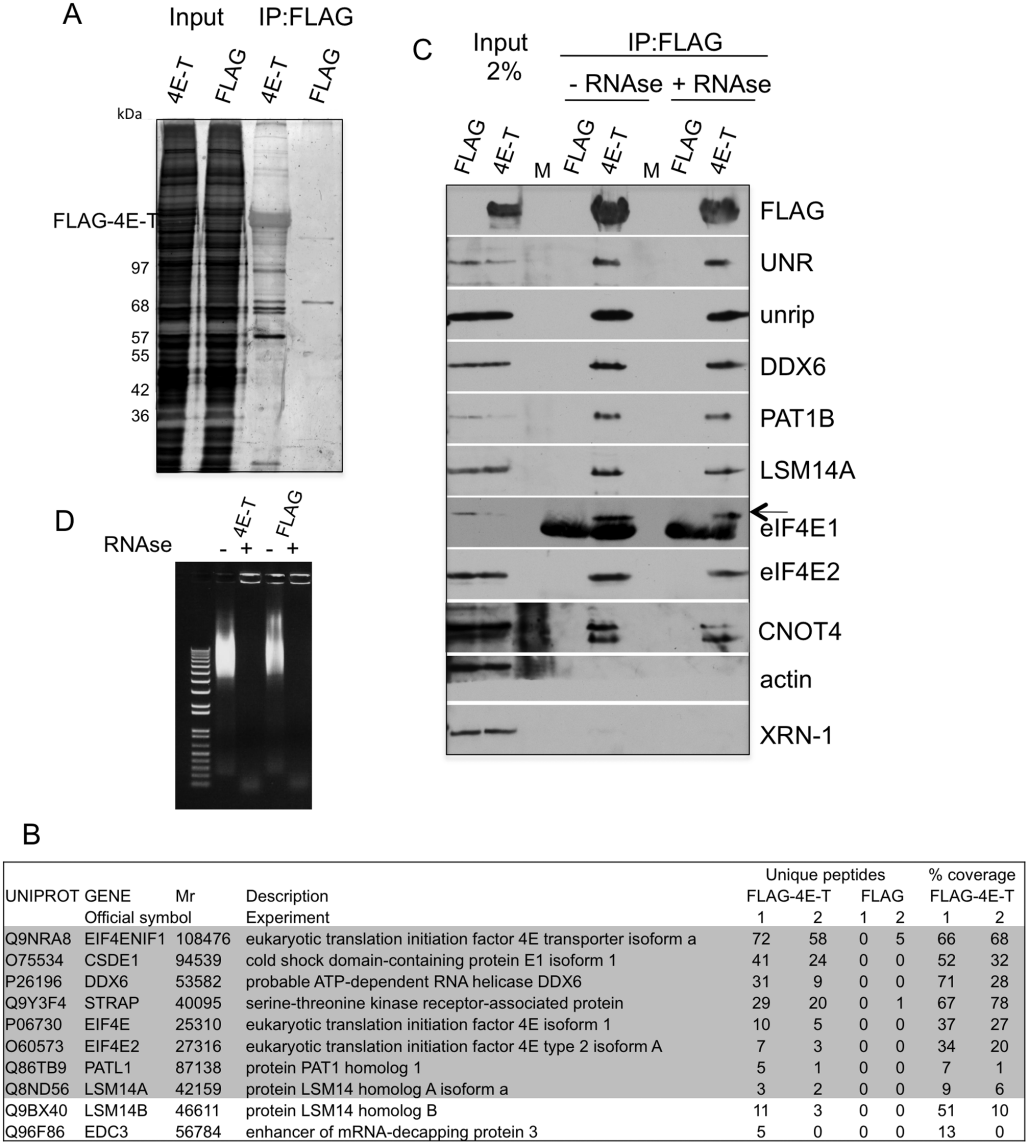
Mass spectrometry of proteins co-immunoprecipitating with FLAG-4E-T. (**A**) Silver-stained gel of input and bound samples obtained in HEK293T cells transfected with FLAG and FLAG-4E-T. Lysates were immunoprecipitated using FLAG-M2-agarose beads and bound proteins eluted with FLAG peptide and analysed by SDS-PAGE. (**B**) Mass spectrometry of immunoprecipitated proteins obtained in two separate experiments (see Materials and Methods). Unique peptide number and % coverage of the top scoring and related proteins is indicated. (**C**) Verification of co-immunoprecipitating proteins by Western blot analysis, with indicated antibodies (corresponding to proteins listed in B with grey background). Lysates were treated or not with RNAse A prior to immunoprecipitation. M indicates lanes with molecular weight proteins standards. (**D**) Agarose gel of Ethidium bromide-stained lysate RNA, before and after RNAse treatment.

To initiate the analysis of how 4E-T represses translation, we determined its protein partners using mass spectrometry, reasoning that these interacting proteins could offer clues to its mechanism of action. FLAG-4E-T or FLAG alone were expressed in HEK293T cells, the resulting lysates were incubated with M2-Sepharose beads, and bound proteins were eluted with FLAG peptide, subsequently analysed by mass spectrometry (Figure 1A). In two independent experiments, approximately two hundred proteins represented by 2 or more unique peptides co-immunoprecipitated specifically with FLAG-4E-T (Supplementary Table S1). GO analysis showed that about 20% of these belong to the mRNA metabolic process category and 30% to the poly(A) RNA-binding function class (*P*-values = 1^−26^ and 4^−42^ respectively). According to the average protein probability scores (PP av, ProteinCenter), the consistent top-scoring proteins binding to 4E-T were the cold-shock domain protein UNR (CSDE1), its interacting partner unrip (also known as STRAP or serine threonine kinase receptor-associated protein) and p54/rck/DDX6 RNA helicase (Figure 1B and Supplementary Table S1). 4E-T also co-immunoprecipitated several P-body proteins in addition to DDX6 including PAT1B and LSM14 proteins, and more weakly, EDC3, judging by peptide number and protein coverage scores. As predicted, FLAG-4E-T also bound eIF4E1 and eIF4E2, two cap-binding proteins known to interact with 4E-T (1,7).

Previously, *Xenopus* 4E-T was shown to co-immunoprecipitate and co-fractionate with Xp54/DDX6, PAT1a and LSM14/RAP55, as well eIF4E1b, an ovary-specific cap-binding protein (16,39,42), demonstrating conserved interactions between 4E-T, these P-body components and cap-binding proteins. However, the binding of human 4E-T to UNR and unrip was not anticipated. UNR, a 95 kDa protein with five RNA-binding cold shock domains performs diverse roles in RNA metabolism, and UNR-deficient mice display embryonic lethality (reviewed (43,44)). Unrip, also essential in mice, is a 38 kDa WD40 protein which tightly interacts in the cytoplasm with UNR (37) and LARP6 (45), and with the SMN/Gemin complex (38,46) required for snRNP biogenesis, in yet to be determined functions (reviewed (47)). Neither protein has to our knowledge been reported to be enriched in P-bodies (see (38,46,48)).

To verify the mass spectrometry interactions, we used western blotting following co-immunoprecipitation from FLAG-4E-T expressing cell lysates, treated or not with RNAse A. As shown in Figure 1C, FLAG-4E-T but not the control FLAG-beads interact with the two eIF4E cap-binding proteins, and with UNR, unrip, DDX6 as well as PAT1B and LSM14A, but not actin nor XRN1 exonuclease. Several of these interactions were subsequently confirmed in reverse immunoprecipitations (see Figure 4B for FLAG-UNR, -unrip and -DDX6). All these interactions were RNA-independent, as they resisted RNase treatment (Figure 1D). Furthermore, the novel 4E-T-UNR RNA-independent interaction was confirmed with endogenous proteins, using both 4E-T and UNR antibodies for immunoprecipitation (Supplementary Figure S1).

We were also interested to assess the binding of CCR4-NOT complex subunits to 4E-T. Previously we showed that GFP-4E-T can interact with FLAG-CNOT1 and FLAG-CNOT7, though binding of these endogenous CNOT subunits was undetectable by western blotting (26). In this study, low levels of CNOT7, CNOT8 and CNOT11 subunit peptides were detected by mass spectrometry (one of each in Experiment 1, data not shown). CNOT4, an evolutionarily conserved E3 ubiquitin ligase (49), was of particular interest as a recent study identified yeast NOT4 as a translational repressor (50). Interestingly, we found that endogenous CNOT4 binds FLAG-4E-T efficiently, also in an RNA-independent manner (Figure 1C).

### Identifying the binding sites of DDX6 and UNR

**Figure 2.**
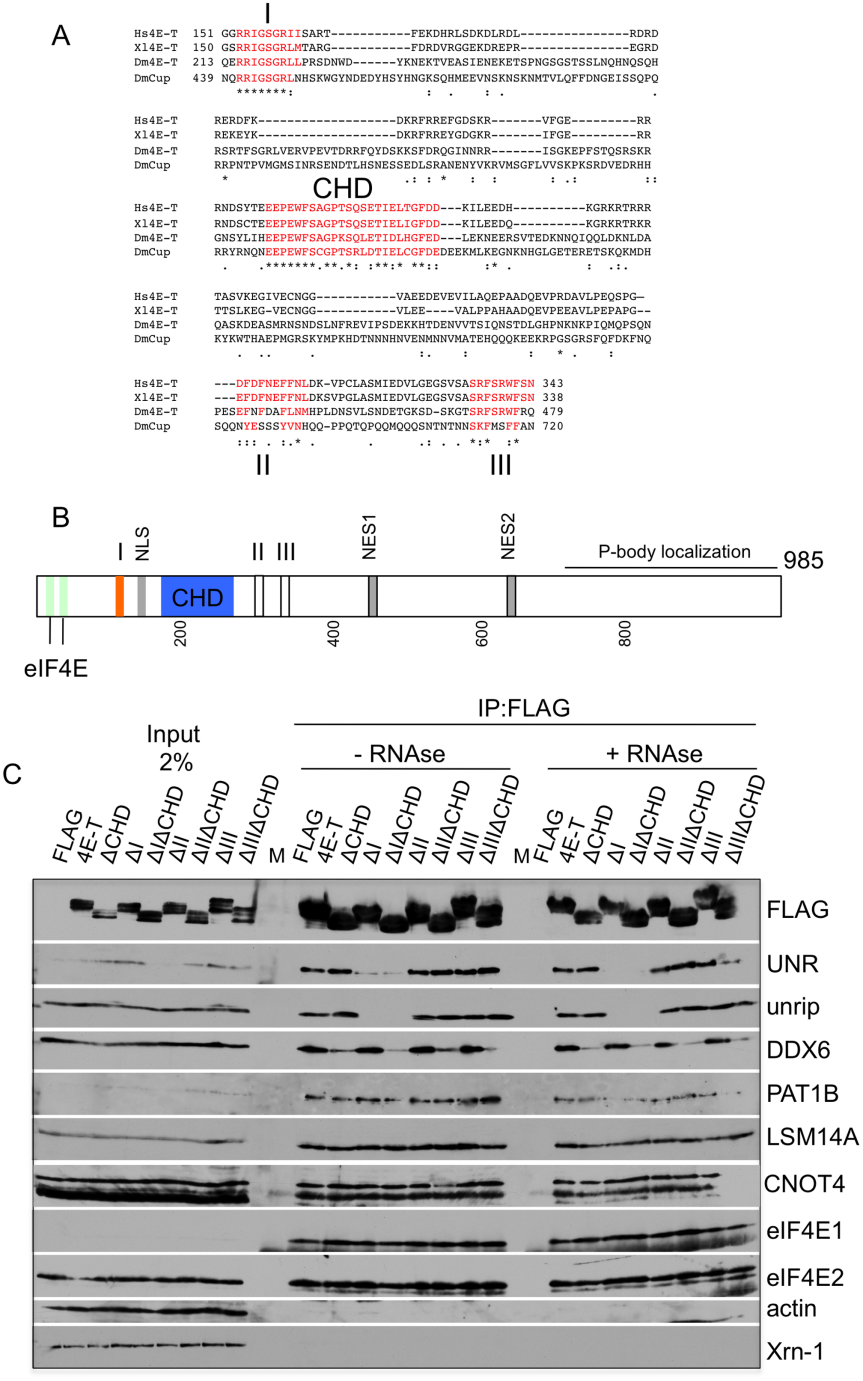
Identifying the binding proteins of motifs I and CHD. (**A**) ClustalW2 alignment of 4E-T and 4E-T related sequences: human (NM_001164501), *X. laevis* (NM_001093241), *Drosophila* Cup (CG11181; NM_078769) and 4E-T (CG32016; NM_166798). The alignment spans residues 152–342 in the human protein; we did not find significant conservation in regions 100–152 and 343–694 in these four proteins. (**B**) Schematic cartoon of human 4E-T, indicating the four conserved motifs I through III and CHD. (**C**) Anti-FLAG immunoprecipitation of untreated and RNAse A-treated lysates from HEK293T cells transfected with FLAG alone, FLAG-4E-T and FLAG-4E-T constructs missing individual motifs I-III and the CHD, or deletion constructs missing combinations of CHD with motif I, or II, or III. Input and bound samples were analysed by Western blotting with indicated antibodies.

The human 4E-T protein is predicted to be largely disordered and to contain low complexity regions, according to http://anchor.enzim.hu and http://www.ncbi.nlm.nih.gov/Structure/cdd/cdd.shtml. When tethered to mRNA, both *Drosophila* Cup and human 4E-T repress translation, without requirement for the N-terminal eIF4E-binding site or the C-terminal P-body localization sequences (26,29). We thus turned to the examination of internal sequences conserved in the vertebrate and *Drosophila* 4E-T/Cup proteins. This revealed a set of four short conserved sequences which we named I-III and CHD ((I (131–161), CHD (208–245), II (291–316) and III (331–346)); Figure 2A and B).

Motifs I and III, while highly conserved between human 4E-T and *Drosophila* Cup, did not resemble to our knowledge any interaction sequences previously characterized in P-body components. On the other hand, motif II, including the sequence FDF, was similar to EDC3, PAT1 and LSM14 protein sequences that interact with DDX6 (51,52). The CHD or Cup homology domain, approximately 25 amino acids long, is the longest most highly conserved sequence common to human 4E-T and *Drosophila* Cup, also present in *C. elegans* IFET-1, but lacks obvious sequence features (26). Given their length and being parts of largely disordered proteins these sequences potentially represent short linear motifs or SliM (53), mediating protein-protein interactions. Notably too, the N-terminal region of 4E-T proteins extending to just downstream of the CHD is enriched in charged amino acids, particularly striking in vertebrate homologues. Indeed, >60% of the residues between motif I and the CHD in human 4E-T comprise Arg, Lys, Glu or Asp, with many in an alternating acidic/basic configuration (Figure 2A). Such ampholyte sequences tend to adopt elongated coil-like conformations, due to the charged residues preference to be solvated (reviewed in (54)).

The four motifs in 4E-T were deleted from the full-length FLAG-tagged protein, singly, and in combination (motifs I–III) with the CHD sequence. The effect of deleting these motifs was then tested in co-immunoprecipitation assays. Strikingly, deletion of motif I suppressed binding of both UNR and unrip to 4E-T, whereas deletion of the CHD sequence prevented binding of DDX6 (Figure 2C). However, deletions of CHD and motif I did not alter the interactions of 4E-T with eIF4E1/2, PAT1B, LSM14A or CNOT4, indicating that they did not result in large scale misfolding (Figure 2C). Furthermore, loss of motifs II and III had no discernible effect on any of the tested co-factors. We concluded that motif I specifically binds UNR/unrip and that the CHD interacts with DDX6. Some residual DDX6 was retained on FLAG-4E-TΔCHD beads, as was also the case for a minor fraction of UNR on FLAG-4E-TΔI beads. While the latter is RNA-mediated, since RNase treatment resulted in full loss of UNR binding, this was not the case for DDX6 (Figure 2C). In the light of documented interactions between DDX6, PAT1 and LSM14 proteins (51,52), it was important to assess whether DDX6, in addition to binding the CHD, may interact with 4E-T indirectly via these P-body components.

### Mapping 4E-T interactions with PAT1B and LSM14A

**Figure 3.**
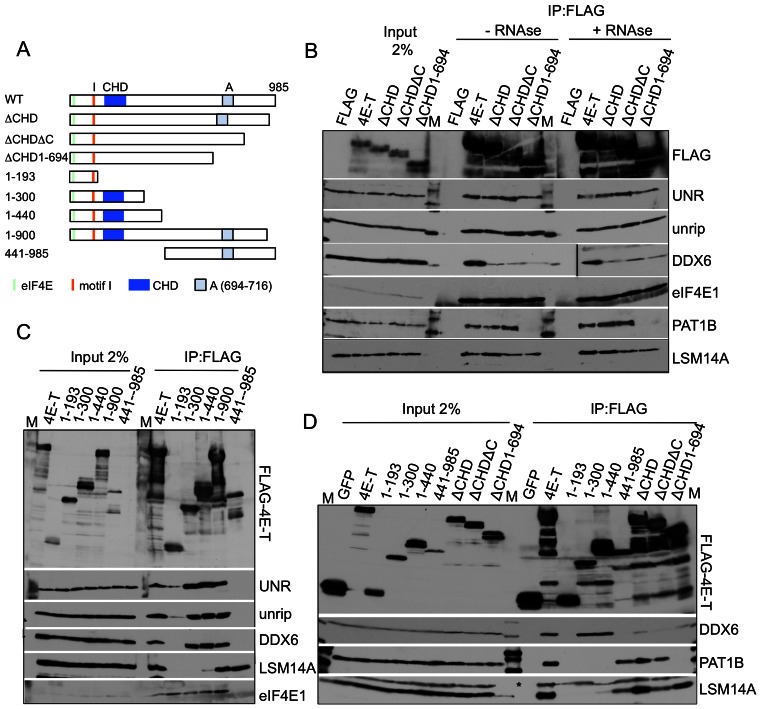
Mapping the binding sites of 4E-T interaction partners PAT1B and LSM14A. (**A**) Schematic cartoon of FLAG-tagged proteins immunoprecipitated in experiments shown in panels B-D. Region A is 694–716, conserved in 4E-T proteins, part of the P- body localization sequences (26). (**B**) 4E-T P-body localization defective 4E-T constructs were combined with deletion of CHD. 4E-TΔC refers to a deletion/truncation mutant of 4E-T: 4E-TΔ694−716(1−845), defective in P-body localization, while 4E-T1-694 is not only P-body localization defective but also dominant negative for endogenous P-bodies (26). Anti-FLAG immunoprecipitations were carried out with FLAG alone, or FLAG-4E-T proteins. Input and bound samples were analysed by Western blot analysis with indicated antibodies. (**C** and **D**) HEK293T cells were transfected with FLAG-4E-T protein plasmids. Anti-FLAG immunoprecipitations were carried out using full-length FLAG-4E-T, and truncated versions, -4E-T1-193, -1-300, -1-440, -1-900 and -441-985 (C), and FLAG-GFP, FLAG-4E-T, -4E-T1-193, -1-300, -1-440, -441-985, -ΔCHD, -ΔCHDΔC and −ΔCHD 1-694. * indicates unrelated protein (D). Input and bound samples were analysed by Western blotting with indicated antibodies.

To determine PAT1B and LSM14A interaction sites, we introduced the ΔCHD deletion into the previously characterized P-body localization defective mutants 4E-TΔC (Δ694−716(1−845)) and 4E-T1-694 (Figure 3A; (26). As expected, DDX6 binding was markedly decreased by the CHD deletion, but not UNR, unrip or eIF4E. PAT1B fails to bind 4E-TΔCHD1-694, but interacts with all other protein mutants, placing its interaction site between amino acids 717 and 845 (Figure 3B). Yet, the same residual binding of DDX6 to 4E-TΔCHD was observed in the absence of PAT1B binding. As LSM14A binding was unaffected in these three protein mutants, we then assessed its binding to truncated fragments of FLAG-4E-T. LSM14A fails to bind the 1–440 fragment, but binds the 1–900 and 441–985 fragments. These and the observations above indicate its interaction site lies between amino acids 441 and 694. As expected, DDX6, UNR and unrip bind to N-terminal 4E-T fragments spanning residues 1–440, but fail to associate with the C-terminal fragment 441–985 (Figure 3C). (We note that binding of UNR and unrip to 4E-T1-193 is specific but partial, possibly reflecting incomplete folding of motif I in this truncation). In contrast, LSM14A and PAT1B bound the C-terminal fragment 441–985 but none of the N-terminal 4E-T fragments (Figure 3C and D). Thus, DDX6 does not appear to stably bind 4E-T via PAT1B or LSM14A, and reciprocally, neither PAT1B nor LSM14A stably interact with 4E-T via DDX6 (Figure 3C and D). Furthermore, these observations suggest that the residual binding of DDX6 to 4E-TΔCHD, rather than resulting from PAT1B/LSM14A-4E-T interactions, may reflect a second minor DDX6 site in the N-terminal half of 4E-T. Alternatively, as 4E-T can self-associate (55), it may result from DDX6 bound by endogenous 4E-T and retained by FLAG-4E-T.

### 4E-T binds UNR rather than unrip

**Figure 4.**
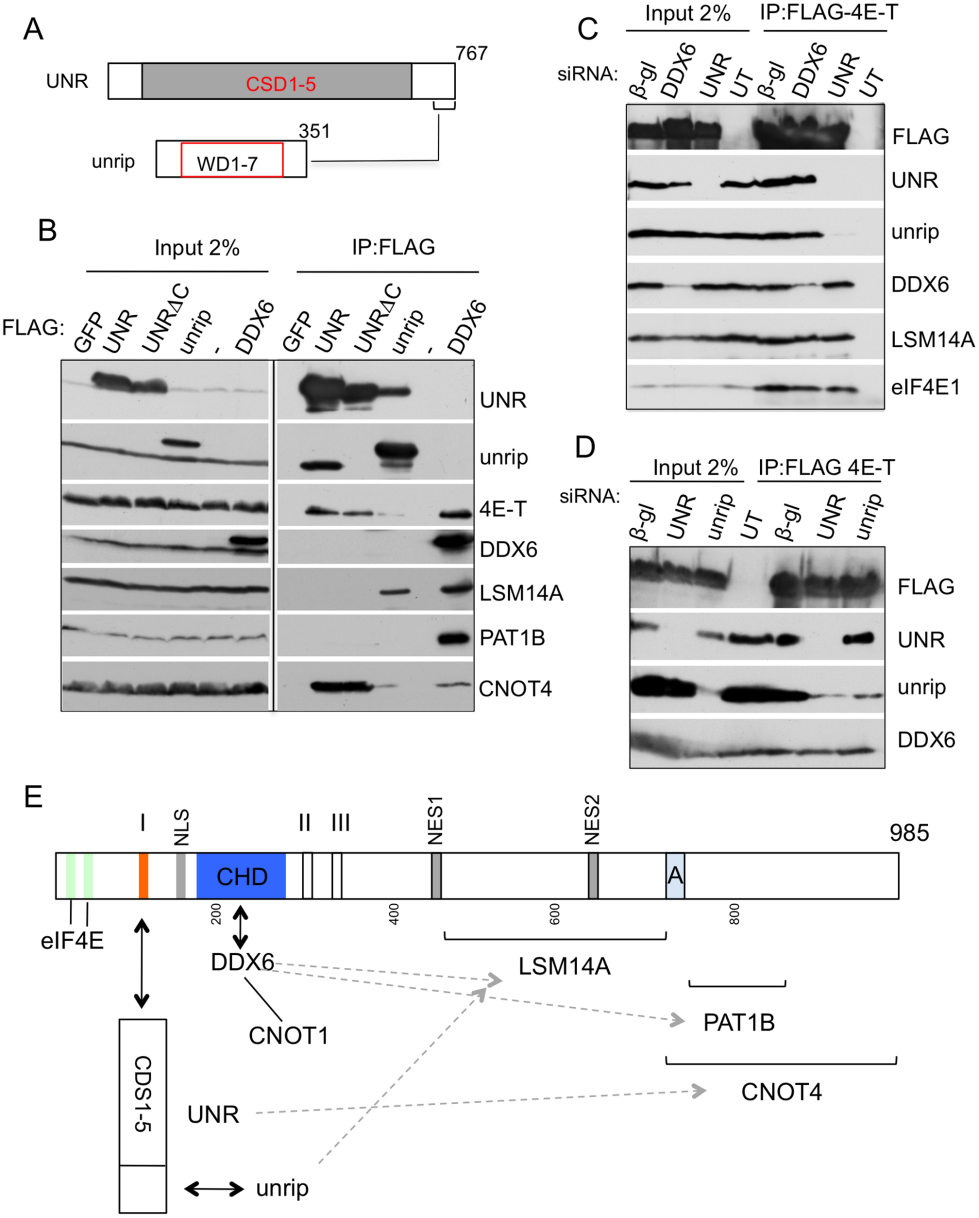
4E-T interacts with UNR, which in turn binds unrip. (**A**) Cartoon of UNR and unrip proteins, indicating their domains and the C-terminal unrip-binding site in UNR. (**B**) Anti-FLAG immunoprecipitations were carried out using FLAG-tagged GFP, -UNR, -UNRΔC, -unrip and -DDX6. UNRΔC is a C-terminally truncated version of UNR predicted not to interact with unrip. UT, untransfected cells. (**C** and **D**) HEK293T were co-transfected with siRNA against β-globin (control), DDX6, UNR or unrip as indicated, and with FLAG-4E-T, or not transfected (UT). Input and bound samples were analysed by western blot with indicated antibodies. (**E**) Schematic cartoon of interaction sites of 4E-T-binding proteins. Region A is 694–716, conserved in 4E-T proteins, part of the P- body localization sequences (26). Interactions shown in this study: solid line—interactions displayed by deletion and truncated fragments of 4E-T, as well as by FLAG-tagged UNR, -unrip and -DDX6. Brackets indicate regions of interactions with indicated proteins displayed by truncated fragments of 4E-T. Dotted grey line, interactions shown by FLAG-tagged UNR, -unrip and -DDX6. Arrows indicate bait identity. Interactions solved structurally in other studies: the canonical and non-canonical 4E-T-eIF4E binding sites (8) and 4E-T-DDX6-CNOT1 (36).

We next examined whether both UNR and unrip bind 4E-T individually or whether one protein bridges the interaction of the other, given their high affinity association (37). First, we took advantage of a recent study showing that unrip interacts with the sequence VLRLPRGPDNTRGF in LARP6, and that a similar C-terminal peptide is present in UNR (^744^VLRQPRGPDNSMGF), downstream of the cold-shock domains (45). We truncated UNR (767 amino acids long) at residue 744 to generate FLAG-UNRΔC, and analyzed its binding to unrip and other proteins (Figure 4B). Indeed, as anticipated, FLAG-UNRΔC fails to immunoprecipitate unrip, but 4E-T binding is not altered, suggesting that the 4E-T-UNR interaction is direct. In contrast, FLAG-unrip interacts very weakly with 4E-T, though this may be mediated by UNR. To test this further, we used siRNAs to deplete UNR or unrip in cells transfected with FLAG-4E-T, as well as control and DDX6 siRNA. Confirming the UNR truncation assay, siRNA-mediated depletion of UNR prevented 4E-T interaction with unrip, while depleting unrip did not affect 4E-T-UNR binding (Figure 4C and D).

We also note that there is no evidence in these pull-down experiments of an affinity between DDX6 and UNR or unrip, though FLAG-DDX6 as predicted from previous work (35,39,51,52) co-immunoprecipitates LSM14A and PAT1B, as well as 4E-T. Moreover, depletion of DDX6 does not affect FLAG-4E-T binding to UNR, and depletion of UNR or unrip does not disrupt FLAG-4E-T interaction with DDX6 (Figure 4B–D). Last, endogenous UNR and DDX6 proteins do not interact (Supplementary Figure S1). We concluded that UNR binds 4E-T directly, while unrip interacts with 4E-T through UNR, and furthermore that there is no detectable binding between DDX6 and UNR, in agreement with the recent DDX6 proteome study (35).

### Multiple interactions between 4E-T interacting proteins: unrip-LSM14A and UNR-CNOT4

In light of the various possible interactions between 4E-T and DDX6, LSM14A and PAT1B, we next examined binding between UNR and unrip and other 4E-T interacting proteins. Unexpectedly, FLAG-unrip, in addition to binding UNR, also co-precipitates LSM14A (Figure 4B). This interaction is not a consequence of unrip binding to UNR, binding in turn to 4E-T, as FLAG-UNR does not co-precipitate LSM14A (Figure 4B). Moreover, FLAG-UNR binds CNOT4 (Figure 4B). This interaction, unlike that of unrip, is independent of the C-terminal sequence as evidenced in UNRΔC, suggesting that UNR could bind simultaneously unrip and CNOT4. Interestingly, FLAG-4E-TΔI, which does not precipitate UNR nor unrip, nevertheless binds to both LSM14A and CNOT4, apparently as well as the full-length protein (Figure 2). Indeed, primary LSM14A binding was mapped to residues 441–694 of 4E-T, rather than motif I, while CNOT4 binding to 4E-T requires its C-terminal 694–985 sequences (Figure 3; Supplementary Figure S2).

Collectively these biochemical observations indicate firstly, the location on 4E-T of UNR/unrip, DDX6, LSM14A, PAT1B and CNOT4 binding sites. Secondly, these observations highlight an additional network of independent interactions between several of these binding proteins, including DDX6-PAT1B, DDX6-LSM14A, unrip-LSM14A and UNR-CNOT4 (summarized in Figure 4E and Supplementary Figure S3)), with such a network likely contributing to the stability of the complex between 4E-T, RNA-binding proteins, eIF4E and mRNA.

### Motif I and the CHD mediate 4E-T repressor activity

**Figure 5.**
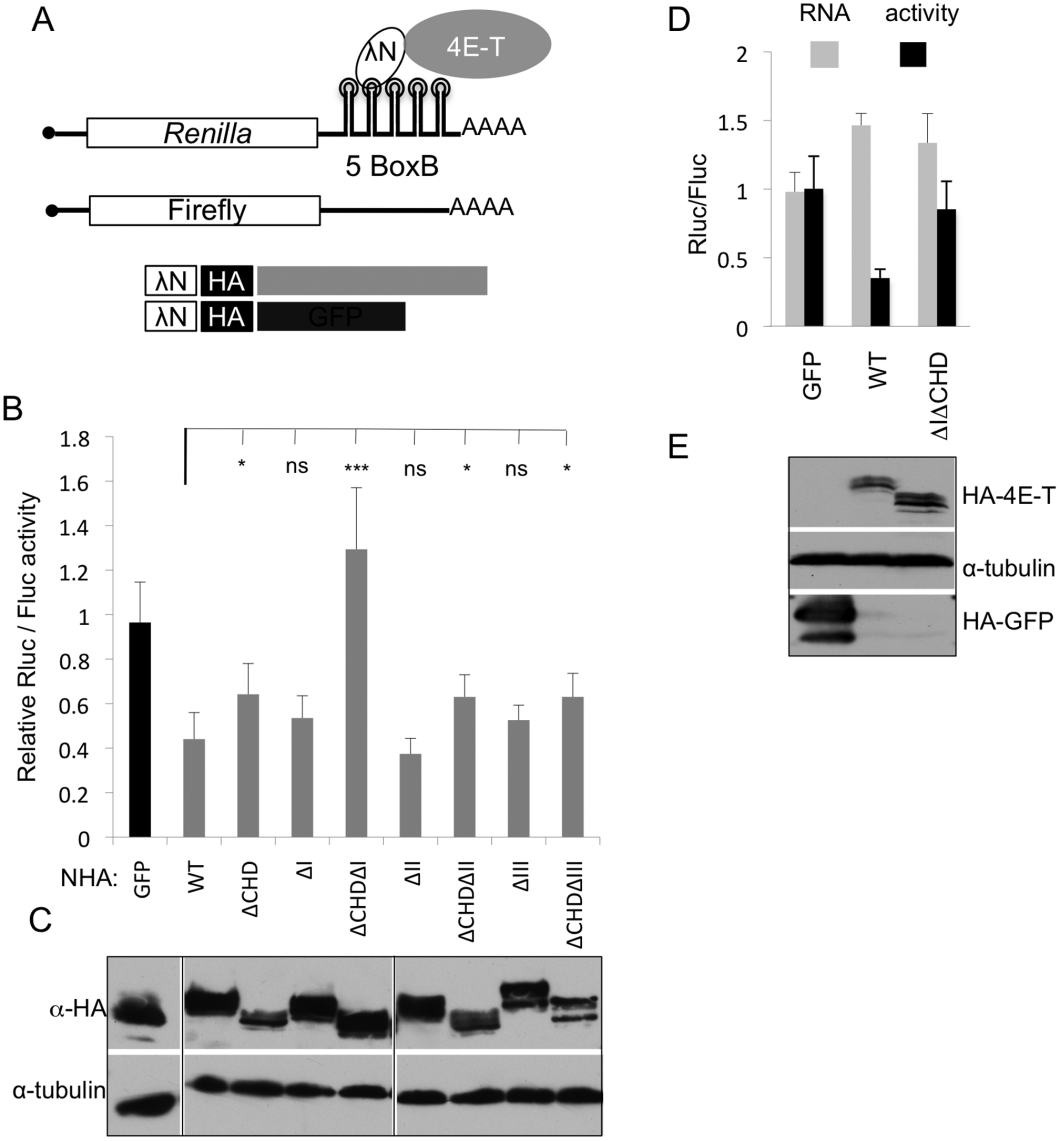
Deletion of both motif I and CHD is required to alleviate translational repression by tethered 4E-T. (**A**) Schematic cartoon of the tether function assay, indicating the luciferase reporter mRNAs, and the λN-HA-tagged (NHA) 4E-T and control GFP proteins. (**B**) The tether function assay with NHA-GFP and NHA-4E-T proteins, full-length (WT) and deletion versions missing the four conserved motifs individually or deletion combinations of motif I-III with CHD (***P < 0.001, *P <0.05, ns not significant, two tailed t test; relative to WT (*n* = 5–6)). (**D**) HEK293T cells were transfected with NHA-GFP, -4E-T and -4E-TΔIΔCHD plasmids and the luciferase mRNA reporter plasmids. The activities of *Renilla* and firefly luciferases were determined by luminometry (black bars), and the levels of their mRNAs (grey bars) by qRT-PCR. (**C** and **E**) Western blots of NHA-tagged proteins from experiments shown in (B) and (D) with indicated antibodies.

To address the possible role of the four conserved motifs in 4E-T mediated translational repression, we used the tether function assay which is particularly powerful in identifying functionally relevant domain(s) in regulatory proteins, including human 4E-T and the related *Drosophila* protein Cup (26,29). In this assay, 4E-T or the control GFP protein, tagged with both a lambda N peptide and an HA epitope, were co-expressed with *Renilla* luciferase mRNA bearing 3′ UTR Box B hairpins and a control firefly luciferase mRNA. Following transfection, lysates were prepared and the relative luciferase activities determined (Figure 5A). Previously, we showed that untethered, HA-tagged 4E-T and GFP did not influence luciferase expression relative to NHA-GFP, and that the 3′ UTR box B hairpins were required for repression by NHA-4E-T (26).

The full-length NHA-tagged 4E-T protein repressed normalized Rluc activity relative to NHA-GFP ((26); Figure 5B). Motifs I-III were deleted from NHA-4E-T, singly, and in combination with the CHD. Interestingly, while deletion of motifs I, II and III individually did not have any significant effect, deletion of the single CHD partially relieved repression (Figure 5B). However, the combined deletion of motif I and the CHD led to complete loss of repression (Figure 5B). This effect was specific as it was not seen with II and CHD or III and CHD double deletions, despite similar levels of expression (Figure 5C). In other words, deleting the CHD consistently led to partial relief of repression, with full relief specifically resulting from the additional deletion of motif I. Importantly, as shown previously (26), repression by tethered 4E-T is at level of translation, not mRNA levels, as determined by qRT-PCR, and lack of repression by 4E-TΔIΔCHD is not due to reporter mRNA stabilization relative to WT 4E-T (Figure 5D. Furthermore, 4E-T represses translation of bound mRNA in a poly(A)-independent manner (Supplementary Figure S4), indirectly supporting the conclusion that tethered 4E-T does not result in decay of bound mRNA. Again, the wild-type and mutant 4E-T proteins were expressed at comparable levels (Figure 5E and Supplementary Figure S4). Finally, wild-type 4E-T protein and 4E-TΔIΔCHD proteins were indistinguishable in terms of cellular distribution, both being cytoplasmic and enriched in P-bodies (see below). In conclusion, we propose therefore that the CHD, aided by motif I, mediates the repression of reporter mRNA by tethered 4E-T.

### ATP hydrolysis by DDX6 contributes to 4E-T mediated repression

**Figure 6.**
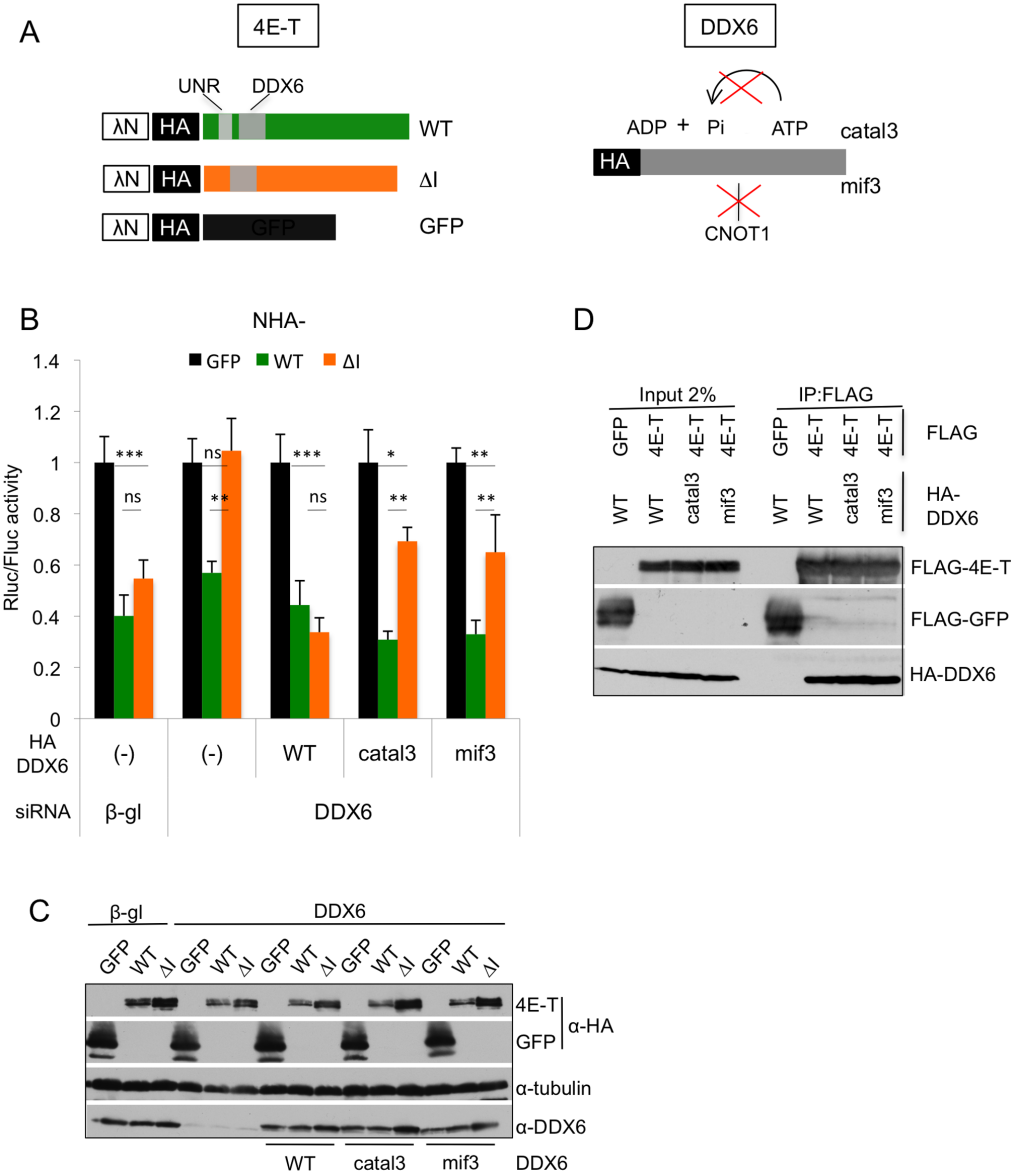
Catalytically active DDX6 mediates 4E-T translational repression. (**A**) Schematic cartoon indicating the components of the combined deletion/depletion tether function assay. (**B**) Tether function assays were carried out with NHA-tagged GFP, full-length 4E-T (WT) or 4E-T lacking motif I,(ΔI) in HEK293T cells co-transfected with control β-globin siRNA, or siRNA against DDX6, and with DDX6 siRNA-resistant plasmids (WT), the catalytically inactive version catal3 and the mutated version mif3, which does not interact with CNOT1. Lysates were analysed by Dual luciferase luminometry. (**P < 0.005, ns not significant, two tailed *t* test; (*n* = 4)). (**C**) Western blot of lysates from transfected cells in (B) analysed with indicated antibodies. (**D**) HEK293T cells were co-transfected with FLAG-GFP or FLAG-4E-T plasmids and with HA-tagged WT and mutant (catal3 and mif3) DDX6. FLAG immunoprecipitations were analysed by Western blotting with FLAG and HA antibodies as indicated.

We next addressed the role of the CHD-binding protein, DDX6, in 4E-T translational repression in a combined depletion/deletion tether function assay (Figure 6A). Here, the repressive effect of tethered 4E-T and 4E-TΔI was tested in cells submitted to DDX6 RNAi (Figure 6B). β-globin siRNA was used as control, and siRNA-mediated DDX6 depletion was shown to be effective by western blotting (Figure 6C). While depletion of DDX6 only modestly relieves repression by NHA-4E-T, it completely suppressed repression by NHA-4E-TΔI (Figure 6B). Therefore, mutation of the CHD or depletion of its ligand DDX6 have the same effect of alleviating repression by tethered 4E-TΔI (Figures 5B and 6B). We made several attempts to similarly combine UNR and unrip depletion to tethering of NHA-4E-TΔCHD, but observed that depleting UNR reduced cell proliferation and adherence, as noted previously by others (56,57), preventing firm conclusions regarding its role (data not shown; see Discussion).

To test the role of DDX6 ATPase activity in repression, we then performed complementation assays with siRNA-resistant wild-type or mutant DDX6 plasmids, bearing mutations in helicase motifs involved in ATP binding and/or hydrolysis (33). Cells were depleted or not of DDX6, and NHA-GFP and globin siRNA were again used as controls. Western blotting verified that the expression of DDX6 plasmid was equivalent, approximating the level of endogenous DDX6 in control cells (Figure 6C). Importantly, repression by tethered NHA-4E-T and NHA-4E-TΔI was fully restored by expressing exogenous wild-type DDX6 (Figure 6B). In contrast, the ATPase-defective DDX6 mutants were inefficient in restoring repression by NHA-4E-TΔI (catal 3, or DEAD→DQAD, Figure 6C; and catal 1, 2 and 4 data not shown). Interestingly, the normally weak ATPase activity of DDX6 is enhanced by CNOT1 (33). Indeed, the DDX6 mif3 mutant (R386E), which is unable to interact with the CNOT1 MIF4G domain (32–34), was also inefficient in restoring repression (Figure 6B). Of note, the catal3 and mif3 mutations did not impair DDX6 binding to 4E-T (Figure 6D). We therefore concluded that the control of DDX6 ATPase activity by CNOT1 contributes to repression by tethered 4E-T.

### The 4E-T-DDX6 interaction mediates microRNA-mediated silencing

**Figure 7.**
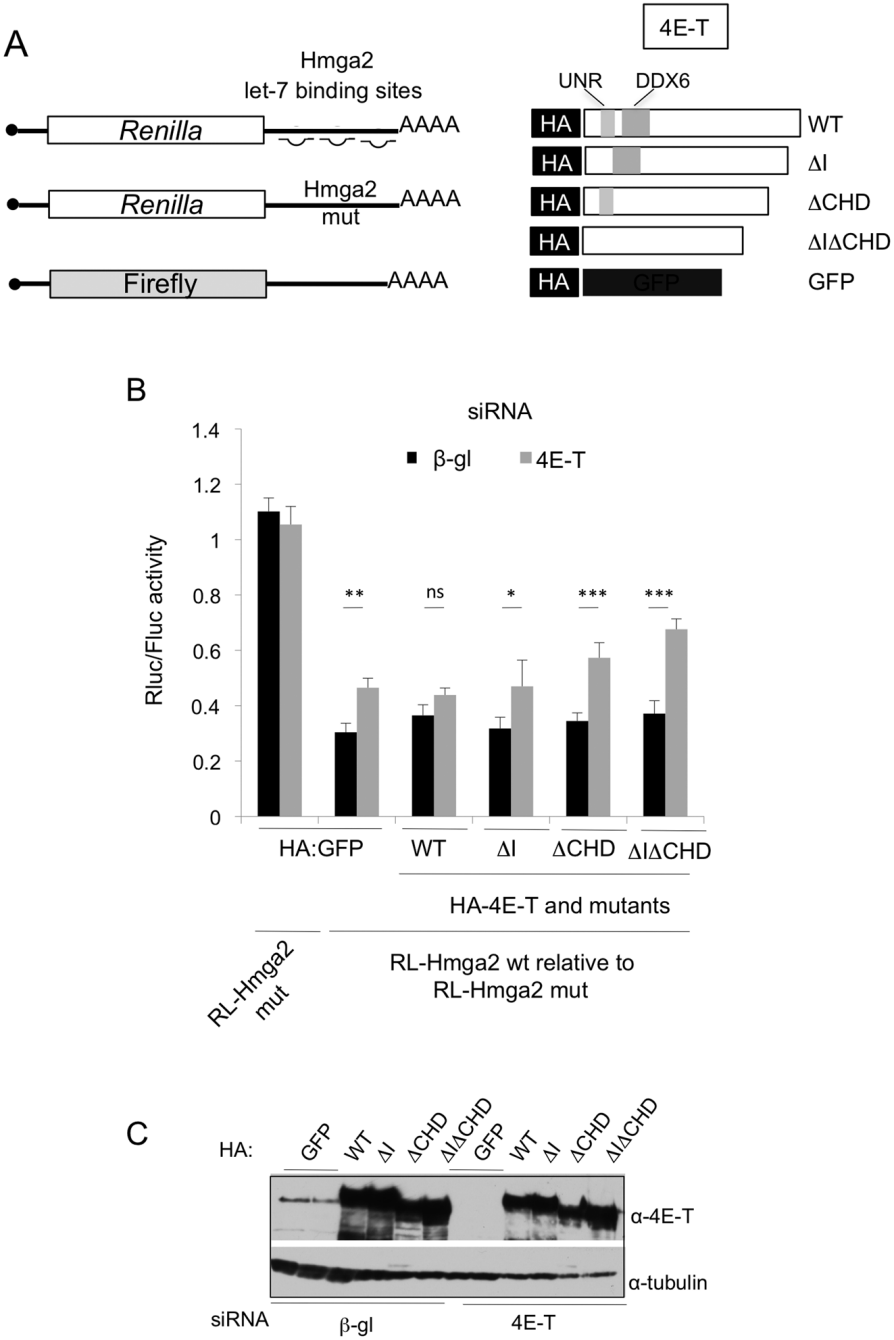
Importance of the DDX6-4E-T interaction for miRNA-mediated repression. (**A**) Schematic cartoon depicting the components of the miRNA reporter assay. (**B**) HeLa cells were treated with control (β-globin) or 4E-T siRNA, and transfected with HA-4E-T (wt and deletion forms), RL-Hmga2 (wt or let-7 binding site) mutant and control FL plasmids. Assays were performed in the presence of the dominant negative PAN2_catal_ and CAF1_catal_ to eliminate effects resulting from mRNA deadenylation (33). Lysates were analysed by Dual luciferase luminometry. (***P < 0.001, *P <0.05, ns not significant, two tailed *t* test; (*n* = 3–4)). (**C**) Western blotting of transfected samples.

To extend the analysis of 4E-T motifs in its regulation of translation, we next turned to a reporter assay based on the 3′ UTR of Hmga2 mRNA which contains seven let-7 sites, as we and others have previously shown the involvement of 4E-T in miRNA silencing (23,26). The assay was adapted from a recent study of DDX6, demonstrating the importance of its ATPase activity and binding to CNOT1 in miRNA-mediated translational repression (Figure 7A; (33)).

Complementation assays were used to test whether 4E-T interactions with UNR and DDX6, or more precisely, its motif I and the CHD, is required for repression of reporter mRNA responding to miRNAs; a mutant reporter with nucleotide substitutions in the let-7 seed regions was used as a control (Hmga2 mut). Following depletion of the endogenous 4E-T by RNAi, cells were transfected with plasmids expressing siRNA-resistant 4E-T, either wild-type or missing motif I, CHD, or both motif I and the CHD, RL-Hmga2 and FL control plasmids as well as PAN2_catal_ and CAF1_catal_. Co-transfection with dominant-negative catalytic mutants of PAN2 and CAF1 permits the detection of miRNA effects at the level of translation, rather than deadenylation ((33) and references therein). Depletion of 4E-T partially relieved repression of RL-Hmga2 reporter mRNA by let-7 miRNA, as previously reported for a reporter mRNA bearing synthetic let-7 binding sites (26). Overexpressed wild-type 4E-T reversed this relief, and relative to WT 4E-T, the three mutant 4E-T proteins were less efficient in repression, though all were similarly expressed (Figure 7B and C). Comparing the mutant proteins, deletion of the CHD had the most significant effect, relative to loss of motif I, and there was no statistically significant difference between relief of repression by ΔCHD and ΔIΔCHD proteins. We conclude therefore that 4E-T mediates miRNA-translational repression primarily via its binding of DDX6 by the CHD, with a minor contribution by motif I.

### The 4E-T-DDX6 interaction is required for P-body assembly

Previously we showed that of several P-body components, DDX6, 4E-T and LSM14A are required for *de novo* P-body assembly in all tested conditions (35), and moreover that assembly depends on DDX6 ATPase activity (39). Indeed, mutating the DDX6 pocket which interacts with LSM14A, PAT1B and EDC3, as well as 4E-T, as shown recently (36), strongly reduced P-body assembly (Mut1 mutant in (35)). To test specifically the importance of 4E-T-DDX6 interactions in P-body assembly, we next assessed the effect of deleting or mutating the DDX6-binding CHD motif in 4E-T.

**Figure 8.**
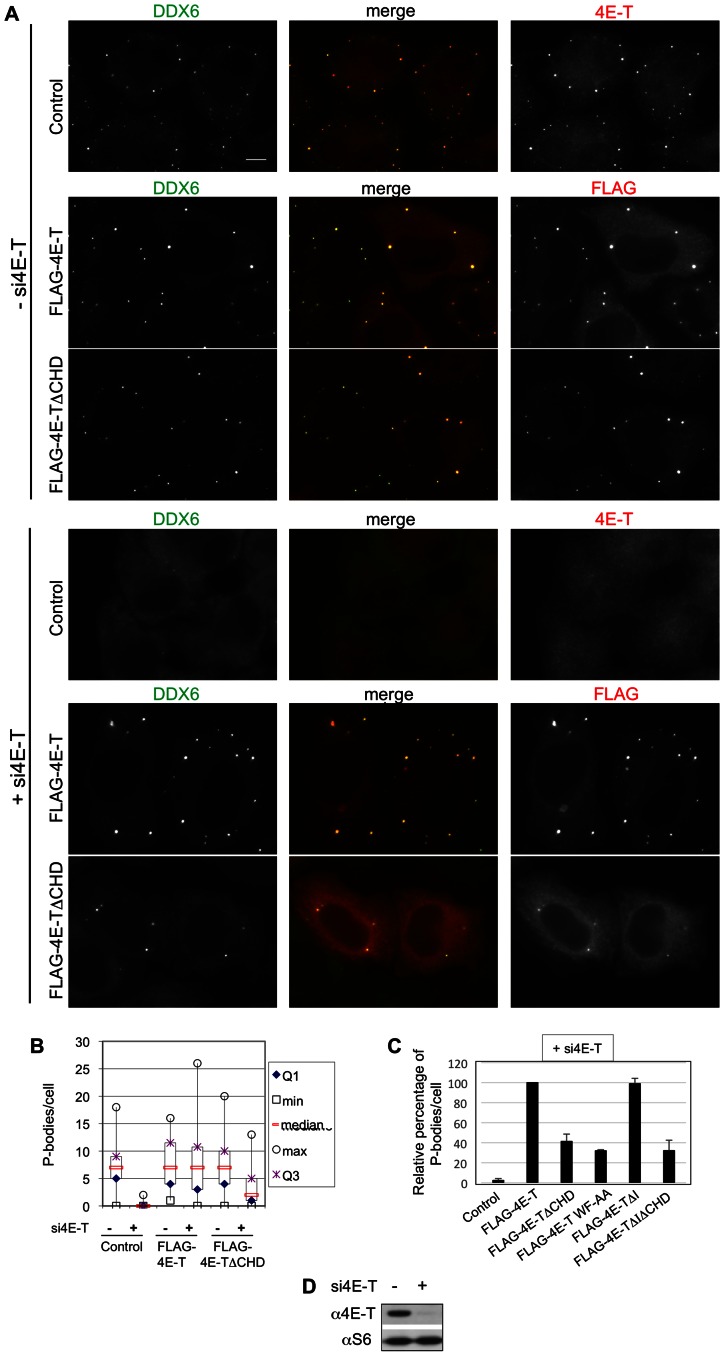
Complementation assay for P-body assembly. (**A**). HeLa cells were successively transfected with no siRNA (upper panels) or siRNA targeting the 4E-T 3′ UTR (lower panels), and 24 h later with no plasmid DNA (control) or FLAG-4E-T plasmid DNA as indicated. After 64 h, cells were analyzed by immunofluorescence using DDX6, 4E-T or FLAG antibodies. Bar, 10 μm. (**B**) Box plots showing the number of P-bodies per cell in the experiment presented in (A). (**C**) Number of P-bodies before and after complementation with indicated FLAG-4E-T proteins. P-bodies were counted in independent experiments (*n* = 2–5, 30–60 cells were counted in each repeat) and expressed as a percentage of P-bodies assembled using wild-type FLAG-4E-T. (**D**) Protein extracts from cells transfected or not with siRNA were analyzed by Western blotting with indicated antibodies.

Hela cells were depleted of endogenous 4E-T by using a siRNA targeting the 3′ UTR of 4E-T mRNA (Figure 8D). After 24h cells were transfected with different FLAG-4E-T plasmids, either wild-type or mutated, and analyzed by immunofluorescence with DDX6 antibody 40 hours after the second transfection. As previously described, 4E-T knock-down disassembles P-bodies in control cells ((35); Figure 8A and B, Supplementary Figure S5). The wild-type 4E-T protein localizes to P-bodies in control cells and allows full complementation of P-body assembly following depletion of the endogenous protein (Figure 8A, Supplementary Figure S5). We next tested the mutated 4E-TΔCHD protein in which the DDX6-binding CHD region is deleted. Interestingly, while 4E-TΔCHD also localized to P-bodies in control cells, it failed to properly restore P-bodies after 4E-T silencing (Figure 8A, B, C, Supplementary Figures S5 and S6). Shown in box plots (Figure 8B) are the number of P-bodies per cell in the experiment presented in Figure 8A, indicating that less than half of P-bodies were detected in this condition. To confirm that P-body assembly was defective, and not just DDX6 recruitment to otherwise intact P-bodies, we also used EDC3 as a second P-body marker (Supplementary Figure S5), and observed a similarly reduced number of P-bodies. These results strongly suggest that DDX6 binding to 4E-T plays an important role in P-body assembly. This was confirmed by transfecting cells with the 4E-T WF-AA plasmid whose mutation in the CHD domain prevents binding of 4E-T to DDX6 (36). Here again, the tagged protein localizes to P-bodies in control cells but was unable to replace the endogenous protein (Supplementary Figure S6). The relative P-body number per cell amounted respectively to 41% and 32% after 4E-TΔCHD and 4E-T WF-AA transfection as compared to wild-type 4E-T (Figure 8C).

Since motif I also contributes to mRNA repression by 4E-T, we asked whether it plays a role in P-body assembly. This is not the case since transfection of 4E-TΔI protein induced 99% P-body assembly as compared to wild type 4E-T (Figure 8C, Supplementary Figure S6A). In addition, the double deletion of motif I and CHD domain (4E-TΔIΔCHD) induced poor complementation for P-body assembly, similarly to 4E-TΔCHD and 4E-TWF-AA (32%, 41% and 32% relative to 4E-T, respectively), and did not show any additive effect of motif I for this process. Of note, the recombinant proteins were expressed at similar levels (Supplementary Figure S6B). Altogether, while all FLAG-4E-T proteins localize to P-bodies, only the ones with a DDX6-binding interface were fully functional for P-body assembly.

## DISCUSSION

Our study extensively characterizes the complex network of multiple interactions between 4E-T and its binding factors, and demonstrates the importance of the DDX6-4E-T interaction in both translational repression and in P-body assembly.

### 4E-T as hub in a complex network of RNA-binding proteins

Mass spectrometry of proteins co-immunoprecipitating with human 4E-T led to the identification of DDX6, UNR/unrip and several P-body proteins involved in mRNA decay and translational repression, including PAT1B and LSM14A, in addition to the cap-binding proteins eIF4E1 and eIF4E2. Previously we showed that in *Xenopus* oocytes, 4E-T and Xp54/DDX6 co-immunoprecipitate with each other alongside PAT1A, RAP55B (LSM14B) and eIF4E1b, so these are conserved interactions (7,16,39). However our new evidence for UNR/unrip binding to human 4E-T was unexpected. We anticipate these interactions to be conserved in *Drosophila* with Me31B/DDX6, UNR and possibly wmd (wing morphogenesis defect)/unrip. Indeed, Cup co-immunoprecipitates with Me31B/DDX6 (29). Moreover, yeast two hybrid screens indicate that *Drosophila* 4E-T also binds Me31B, as well as UNR (58). However, *C. elegans* does not possess UNR (43) nor unrip genes, according to database searches.

Subsequent mapping of these protein binding sites led to the conclusions summarized in Figure 4 and Supplementary Figure S3. First, DDX6 interacts with the CHD motif shared by vertebrate, fly and worm 4E-T homologues, in agreement with recent *in vitro* and *in vivo* data (23,36). Secondly, motif I in human 4E-T binds UNR, which in turn interacts with unrip. Motif I is conserved in vertebrate and fly proteins, but, interestingly, is absent in *C. elegans* IFET-1, accompanied by the lack of UNR/unrip proteins. Third, LSM14A binds 4E-T sequences spanning residues 440–694 while PAT1B interacts with residues 717–845, and CNOT4 with sequences downstream of residue 694.

We also evidence novel additional interactions between 4E-T co-factors including UNR-CNOT4, presumed to be mutually exclusive with 4E-T-CNOT4 binding, as deleting the UNR-binding site in 4E-T does not reduce 4E-T interaction with CNOT4. Intriguingly, 4E-T-CCR4-NOT complex binding is enhanced when the eIF4E-binding site in 4E-T is mutated, as shown for FLAG-CNOT1, FLAG-CNOT7 and endogenous CNOT4 subunits, correlating with increased decay of tethered reporter mRNA ((26); this study, Supplementary Figure S2). As a RING E3 ligase, one proposed role for NOT4 is ubiquitination of stalled peptides (reviewed in (59)). Notably, yeast NOT4 and Dhh1/DDX6 are required for translational repression of transcripts that cause transient ribosome stalling (50). Unlike in yeast, CNOT4 is not a core subunit of the CCR4-NOT complex in human cells. Indeed, metazoan CNOT4 lacks the high affinity residues required to bind CNOT1 (49), explaining its weaker *in vivo* association (60). In view of the well-characterized interaction between DDX6 and CNOT1 (32–34,61), 4E-T may serve as an intermediary link between CNOT1 and CNOT4 subunits.

Altogether then, 4E-T networks a complex set of interactions with several RNA-binding proteins (DDX6, PAT1B, LSM14A, UNR), the cap-binding eIF4E proteins and unrip. Our current list is no doubt incomplete and only reports stable interactions; transient ones with additional proteins including TTP have been documented (23). Plausibly, these multiple interactions serve to crosslink 4E-T complexes to form higher order assemblies, such as RNP granules.

### The roles of the CHD/DDX6 and motif I/UNR in 4E-T mediated translational repression

We provide evidence that the CHD and I motifs together repress translation of mRNAs bound by 4E-T. The CHD motif mediates DDX6 binding, and the ATPase activity of DDX6 and its interaction with CNOT1 are required for its repressive function. However, the role of DDX6 in 4E-T mediated repression could only be revealed when motif I was deleted from 4E-T. Indeed, in agreement with our work, depleting *Drosophila* Me31B/DDX6 did not relieve repression of reporter mRNA by tethered Cup (29). Motif I binds UNR/unrip; which are major interacting factors of 4E-T. In addition to mediating repression in the tether function assay, the CHD, here with a reduced contribution of motif I, also contributes to translational repression of a miRNA reporter mRNA by 4E-T.

In yeast, DDX6/Dhh1 has been characterized both as a repressor of translation, and as an enhancer of decapping (62,63). We currently understand metazoan DDX6 to act principally as a translational repressor, the conclusion being supported by genetic ablation approaches in *Drosophila* oocytes (64), tether function assays in *Xenopus* oocytes (65), and depletion and reporter studies in mammalian cells (33,66,67).

Recent structural and functional studies have highlighted the critical role of DDX6 in microRNA-mediated repression, by interacting with the MIF4G domain of CNOT1, a key effector of miRNA functions downstream of GW182/TNRC6 proteins (32–34,61). 4E-T also contributes to miRNA-mediated repression, as its depletion alleviates silencing of let-7 target mRNAs, albeit modestly (this study, (23,26,61)). Our evidence for the 4E-T-DDX6-CNOT1 interaction being important for translational repression further highlights the possible role of 4E-T in miRNA repression. The 4E-T-DDX6-CNOT1 axis was also implicated in translational repression in *Xenopus* oocytes, using tethered CNOT1 impaired for both DDX6 and 4E-T binding (30). As CNOT1 participates in several specific gene silencing pathways, for example via TTP (68) and Nanos (69), the 4E-T–DDX6–CNOT1 interactions may also regulate their target mRNAs. 4E-T protein levels are considerably lower than those of DDX6 (70), and from this simple perspective it would appear unlikely that it could regulate a substantial population of mRNAs. However, in view of the potential of DDX6 to oligomerize on mRNA (42), while multiple DDX6 molecules may associate with an individual mRNA, just one complexed with 4E-T and CNOT1, we speculate, could impact mRNA translation.

Our mapping and functional data are supported by recent structural evidence for the interaction of the CHD motif of 4E-T with DDX6 (36). Interestingly, the CHD peptide binds to the same DDX6 region as do PAT1, EDC3 and LSM14, implying mutually exclusive interactions of these proteins with DDX6 (51,52). Strikingly, only 4E-T, and not PAT1 nor EDC3, can associate with DDX6 bound to CNOT1 (36), suggestive of a possible temporal relay of interactions mediating first translation repression (CNOT1–DDX6–4E-T) followed by mRNA decapping and decay (DDX6-PAT1/EDC3).

As stated above, we postulate that UNR is responsible for motif I function. Supporting this possibility, UNR has been shown to regulate translation (37,71–73), and mRNA decay (73,74). In the case of *Drosophila* msl-2 mRNA, UNR and the RNA-binding protein Sxl, interacting with separate nearby msl-2 3′ UTR binding sites, prevent 43S ribosome binding (73). Interestingly, UNR and Sxl do not interact with each other, but rather mediate an intertwined cooperative recognition of msl-2 RNA, forming a ternary complex (75). During *Drosophila* dosage compensation, UNR facilitates the interaction of the RNA helicase MLE and the long non-coding RNA roX2, but in this case UNR interacts with MLE directly, stimulated by roX2 RNA (76). UNR and the DDX6 helicase do not bind each but occupy nearby sites in 4E-T, rather than in regulated mRNA as in the case of msl-2. Our data suggest that the two proteins may act redundantly to repress translation, or to affect different stages of translation. However, we cannot exclude alternative possibilities for the role of motif I including: (i) that its deletion from 4E-T prevents the binding of another co-repressor, yet to be identified, (ii) that deletion of motif I disturbs the ampholyte property of this 4E-T region and impacts its association with co-factors indirectly or (iii) that its deletion disrupts a potentially stabilizing complex network of multiple interactions important for translational repression.

Interestingly, according to Genome Browser and UniProt, an alternatively spliced isoform of human 4E-T (variant 2; Q9NRA8-2), 811 amino acids long, lacks both exon 6 and 7. Significantly, as exon 6 encodes motif I and exon 7 the CHD as well as the NLS, the skipped isoform is predicted to be only cytoplasmic, not to repress translation of associated mRNAs, but nevertheless bind eIF4E and reduce its available levels. Indeed, isoform-specific qRT-PCR in human tissue samples supports the existence of this skipped variant (Supplementary Figure S7), providing a potential example of alternative splicing of intrinsically disordered regions ‘rewiring’ protein interactions (77,78), and future studies addressing its expression and regulation is of considerable interest.

### The DDX6–4E-T interaction is also important for P-body assembly

While mammalian P-bodies remain to be purified, they are known from immunostaining and fluorescent protein tagging to contain dozens of RNA-binding proteins, including decay enzymes, translational repressors, one sole translation initiation factor, eIF4E and 4E-T; many of these being conserved in germline RNP granules. Silencing of several of these P-body components, including GW182, LSM1, DDX6, 4E-T, LSM14A, EDC4, CPEB1 and PAT1B (reviewed in (35)), leads to a reduced number of P-bodies or their disappearance, indicating the participation of multiple proteins in P-body maintenance. A recent *de novo* P-assembly study, based on the silencing approach coupled with the induction of P-bodies in various conditions concluded that in addition to DDX6 (39,79), 4E-T and LSM14A were absolutely required for the formation of full-size P-bodies, while PAT1B and EDC3 were dispensable (35). In the light of the subsequently solved structure of DDX6 with the CHD peptide, these observations suggest that of these four mutually exclusive DDX6 partners (36,51,52), the association of either 4E-T or LSM14A to the common interface on DDX6 leads to P-body assembly. We substantiated this hypothesis here by reintroducing 4E-T proteins to silenced cells, and showed that preventing DDX6 binding to 4E-T by deletion of the CHD or mutation of critical binding residues substantially decreased P-body assembly relative to full-length 4E-T. On the other hand, motif I was dispensable for P-body formation, which is consistent with the absence of UNR/unrip from P-bodies.

In conclusion, we provide three independent lines of experimental evidence supporting the importance of the DDX6-4E-T interaction in mRNA silencing. Our biochemical mapping and functional assays demonstrate that the DDX6-4E-T interaction mediates both translational repression (in the tether function assay and miRNA reporter assay) and *de novo* P-body assembly, suggesting that these processes are intimately linked.

## Supplementary Material

SUPPLEMENTARY DATA
