# Supplementary material for: The DDX6–4E-T interaction mediates translational repression and P-body assembly
Source: Nucleic Acids Res. 2016 Jun 24;44(13):6318–34. doi: 10.1093/nar/gkw565 (PMC5291280; doi:10.1093/nar/gkw565)
Supplement: SUPPLEMENTARY DATA [file supp_44_13_6318__index.html]

The DDX6–4E-T interaction mediates translational repression and P-body assembly — The DDX6–4E-T interaction mediates translational repression and P-body assembly — SUPPLEMENTARY DATA 

# The DDX6–4E-T interaction mediates translational repression and P-body assembly

## SUPPLEMENTARY DATA

- SUPPLEMENTARY DATA
