# Supplementary material for: The DDX6–4E-T interaction mediates translational repression and P-body assembly
Source: Nucleic Acids Res. 2016 Jun 24;44(13):6318–34. doi: 10.1093/nar/gkw565 (PMC5291280; doi:10.1093/nar/gkw565)
Supplement: SUPPLEMENTARY DATA [file supp_gkw565_nar-00687-v-2016-File010.pdf]

Supplementary material

The DDX6-4E-T interaction mediates translational repression and P-body assembly

Anastasiia Kamenska, Clare Simpson, Caroline Vindry, Helen Broomhead, Marianne Bénard, Michèle Ernoult-Lange, Ben P. Lee, Lorna W. Harries, Dominique Weil and Nancy Standart

Inventory of Supplemental data:

Supplementary Materials and Methods

Supplementary Figure and Table legends

Supplementary References

Supplementary Figures and Table

Suppl. Fig. 1, relates to Fig. 1
Suppl. Fig. 2, relates to Figs. 2 and 3
Suppl. Fig. 3, relates to Fig. 4
Suppl. Fig. 4, relates to Fig. 5
Suppl. Fig. 5, relates to Fig. 8
Suppl. Fig. 6, relates to Fig. 8
Suppl. Fig. 7
Suppl. Table, relates to Fig. 1

Supplementary Materials and Methods

Immunoprecipitation of endogenous 4E-T and UNR

For immunoprecipitation of endogenous 4E-T, 5 mg HEK293 cell lysate protein in NET buffer supplemented with 0.2 %gelatin were incubated at 4°C for 1 hour with 13 μl of rabbit or goat anti-4E-T antibodies or no antibody (control), and then 120 μl of protein G Sepharose beads were added. After an overnight incubation with constant rotation at 4°C, beads were washed and associated proteins were eluted in SDS sample buffer. Immunoprecipitated proteins were migrated along with 30 μg of lysate. The western blot was developed with rabbit anti-4E-T, -UNR and –unrip antibodies.

For immunoprecipitation of endogenous UNR, 2.5 mg of HEK293 cell lysate protein in FLAG IP buffer supplemented with 20 μg/ml RNase A (as in Fig. 1) were incubated at 4°C for 2 hours with 15 μg of rabbit anti-UNR antibodies (Novus) or no antibody (control), and then 50 μL of Dynabeads Protein A magnetic beads (Life Technologies, France) were added. After 2 hours at 4°C with constant rotation, beads were washed, and associated proteins were eluted in SDS sample buffer. Immunoprecipitated proteins were migrated along with 30 μg of lysate. The western blot was developed with rabbit anti-UNR antibodies and -4E-T antibodies.

GFP-Trap immunoprecipitation

These binding assays were described previously (1).

Quantitative real-time PCR

Quantitative real-time PCR assays specific to wild-type human 4E-T (NM_019843) and exon 6/7 skipped (NM_001164502) isoforms were designed (Life Technologies). Assays were optimized over 7 serial 1:2 dilutions of pooled cDNA. 100 ng of commercially-sourced, tissue-specific RNA

(Becton, Dickinson & Co., Franklin Lakes, NJ, USA: BioChain Institute, Newark, CA, USA: Ambion®) was reverse transcribed using SuperScript® VILO™ Master Mix (Life Technologies) in 20 μl reactions, according to the manufacturer's instructions. 0.5 μl cDNA was added to a 5 μl qRT-PCR reaction including 2.5 μl TaqMan® Universal Master Mix II, no UNG (Life Technologies, Foster City, CA, USA) and 0.25 μl probe and primer mix (corresponding to 900 nM each primer and 250 nM probe; probe and primer sequences are available on request). Reactions were run in quadruplicate on the 7900HT Fast Real-Time PCR System (Life Technologies, Foster City, CA, USA). Amplification conditions were a single cycle of 95°C for 10 minutes followed by 45 cycles of 95°C for 15 seconds and 60°C for 1 minute. The amount of wild-type 4E-T and exon 6/7 skipped isoforms was then quantified by isoform-specific qRT-PCR in tissue samples relative to the three endogenous control genes: beta 2 microglobulin (B2M), beta-glucuronidase (GUSB) and glyceraldehyde 3-phosphate dehydrogenase (GAPDH), which were empirically validated not to vary across the tissues tested. Quantifications were carried out by the Comparative Ct approach, and data were normalized to the median value of the wild-type isoform across all tissues tested.

Supplementary Figures and Table Legends

Suppl. Figure 1

Co-immunoprecipitation of endogenous 4E-T, UNR and unrip proteins. A. Endogenous 4E-T co-precipitates with UNR and and unrip**.** HEK293 cell lysates were incubated with anti-4E-T antibodies (goat and rabbit) and Protein G Sepharose. Bound proteins were analysed along with input lysate and control beads only by western blotting, using indicated antibodies. **B. Endogenous UNR co-precipitates 4E-T.** HEK293 cell lysates were supplemented with RNAse A as in Fig. 1 and incubated with anti-UNR antibodies and Protein A Dynabeads. Bound proteins were analysed along with input lysate and control beads only by western blotting, using indicated antibodies.

Suppl. Figure 2

GFP-4E-T interacts with CNOT4. HEK293 cells were transfected with GFP, GFP-4E-T, GFP-4Emut (4E-T mutated in its eIF4E-binding site; (1)) and GFP-4E-T1-694, and lysates were submitted to GFP-Trap immunoprecipitation. Input and bound samples were separated by a denaturing gradient gel, and analysed by western blotting with indicated antibodies. M is a molecular weight protein standard.

Suppl. Figure 3

Schematic cartoon of interaction sites of 4E-T-binding proteins, summarizing data shown in Figs. 2, 3 and 4.

Suppl. Figure. 4

4E-T represses translation of bound mRNA in a poly(A)-independent manner. A. Schematic cartoon of the tether function assay, indicating the polyadenylated and non-polyadenylated

luciferase reporter mRNAs (where the SV40 polyadenylation signal is replaced with a histone stem-loop hairpin and a hammerhead ribozyme (2), and the λN-HA-tagged (NHA) 4E-T and control GFP proteins. **B.** Repression by 4E-T does not require bound mRNA to be polyadenylated. Ratios of *Renilla* and firefly luciferase activities are shown. **C.** Western blotting of NHA-tagged proteins with indicated antibodies.

Suppl. Figure 5

P-body analysis using EDC3 as a P-body marker

A duplicate sample of cells presented in Figure 8A was analyzed by immunofluorescence using EDC3, 4E-T or FLAG antibodies. Cells were first transfected with no siRNA (upper panels) or with siRNA targeting 4E-T (lower panels), and 24 hours later with no plasmid DNA (control) or FLAG-4E-T plasmid DNA as indicated. Bar, 10 μm.

Suppl. Figure 6

Complementation assay with various FLAG-4E-T mutants

A. Cells were successively transfected with no siRNA (upper panels) or siRNA targeting 4E-T 3'UTR (lower panels), and 24 hours later with different FLAG-4E-T plasmid DNA as indicated. After 64 h, cells were analyzed by immunofluorescence using DDX6, 4E-T or FLAG antibodies. Bar, 10 μm. **B**. **Western blot analysis of transgene expression.** Protein extracts from cells transfected with FLAG-tagged plasmids were analyzed by Western blotting with indicated antibodies.

Suppl. Figure 7

A. Schematic cartoon of the human EIFENIF1/4E-T gene exons 5-8, and skipping of exon 6 and 7 in human 4E-T variant 2. Not to scale. **B. The expression of wild-type and exon 6/7 skipped transcripts of EIF4ENIF across a panel of human tissues**. Expression levels are given

relative to the endogenous controls B2M, GAPDH and GUSB, and are normalised to the median levels of full length EIFENIF1 transcripts in all tissue types. Expression of the full-length mRNA varies considerably, with highest levels seen in brain, and the extent of skipping varies across tissues too. According to Uniprot, the skipped 4E-T variant two is conserved in primates and rodents. Error bars represent the range of expression as calculated from the standard deviation of measurement.

Suppl. Table 1

Full list of 4E-T-interacting proteins. Shown are data from two separate mass spectrometry experiments, each with a FLAG-4E-T protein and FLAG-only control. The results, ranked by peptide number in experiment 1, were filtered as follows – any proteins with just one peptide in both experiments were discarded, as were proteins with peptides in control samples only, and the proteins with a significant number of peptides (approx >30%) in control samples including heat shock proteins, ribosomal proteins, tubulin, actin etc. Exceptionally, the data for PABPC1 and PABPC4 was retained, in view of the possible connection with UNR (see Discussion). Definitions: PP av - Protein probability score; Unique peptide sequences (ignoring modified ones) for all proteins of the gene; EMPAI Calc Exponentially Modified Protein Abundance Index - calculated by ProteinCenter.

References

1. Kamenska, A., Lu, W.-T., Kubacka, D., Broomhead, H., Minshall, N., Bushell, M. and Standart, N. (2014) Human 4E-T represses translation of bound mRNAs and enhances microRNA-mediated silencing. *Nucleic Acids Res*, **42**, 3298-3313.
2. Chekulaeva, M., Mathys, H., Zipprich, J.T., Attig, J., Colic, M., Parker, R. and Filipowicz, W. (2011) miRNA repression involves GW182-mediated recruitment of CCR4-NOT through conserved W-containing motifs. *Nat Struct Mol Biol.*, **18**, 1218-1226.

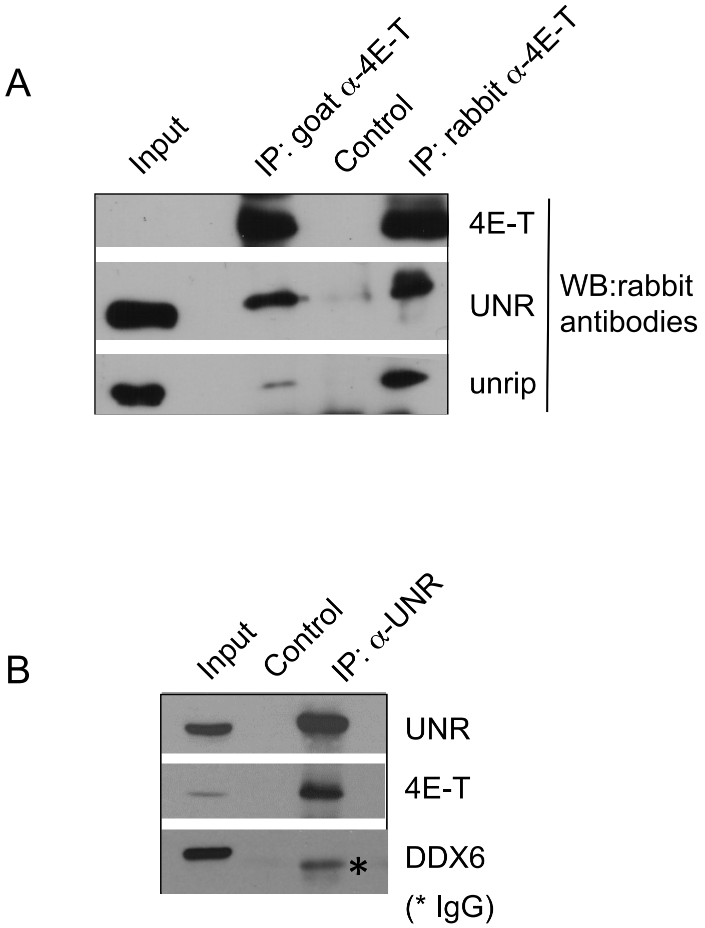
A
Input
IP: goat α-4E-T
Control
IP: rabbit α-4E-T
4E-T
UNR
unrip
WB:rabbit antibodies
B
Input
Control
IP: α-UNR
UNR
4E-T
DDX6
*
(* IgG)

Suppl. Fig. 1

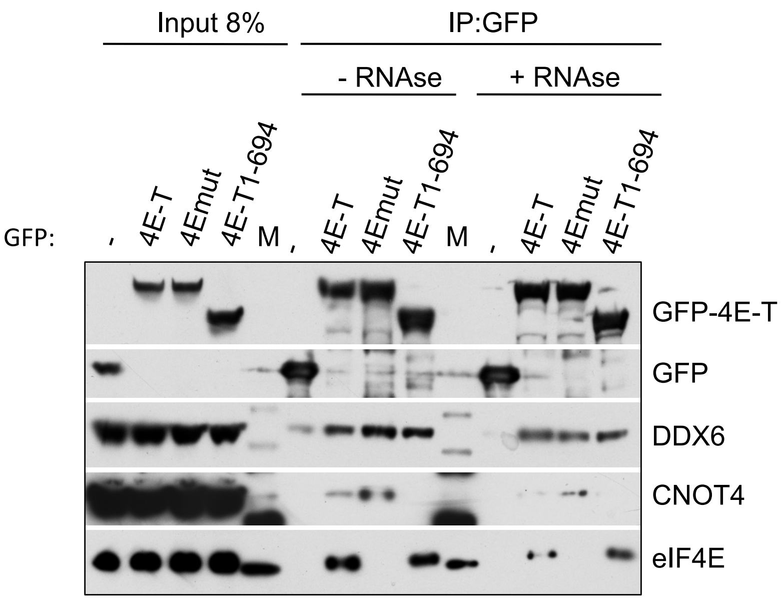
Input 8%
IP:GFP
- RNAse
+ RNAse
GFP:
-
4E-T
4Emut
4E-T1-694
M
-
4E-T
4Emut
4E-T1-694
M
-
4E-T
4Emut
4E-T1-694
GFP-4E-T
GFP
DDX6
CNOT4
eIF4E

Suppl. Fig. 2

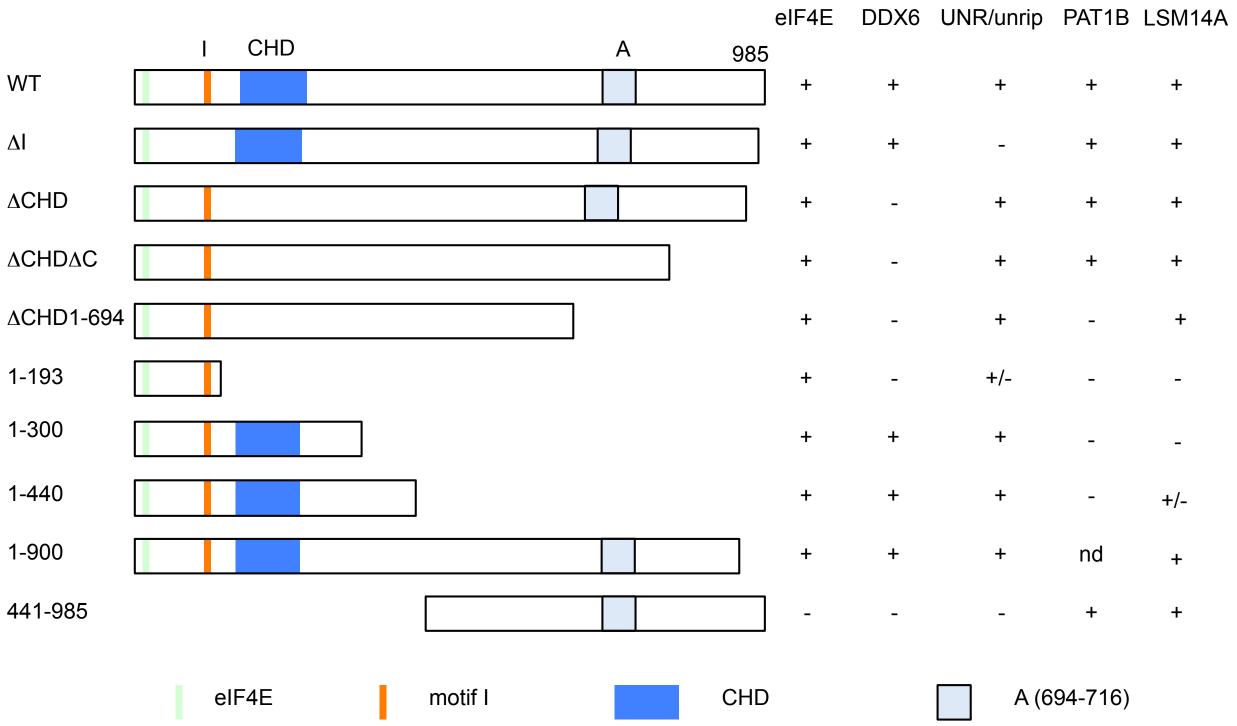
eIF4E DDX6 UNR/unrip PAT1B LSM14A
I CHD A 985
WT + + + + +
ΔI + + - + +
ΔCHD + - + + +
ΔCHDΔC + - + + +
ΔCHD1-694 + - + - +
1-193 + - +/- - -
1-300 + + + - -
1-440 + + + - +/-
1-900 + + + nd +
441-985 - - - + +
eIF4E
motif I
CHD
A (694-716)

Suppl. Fig. 3

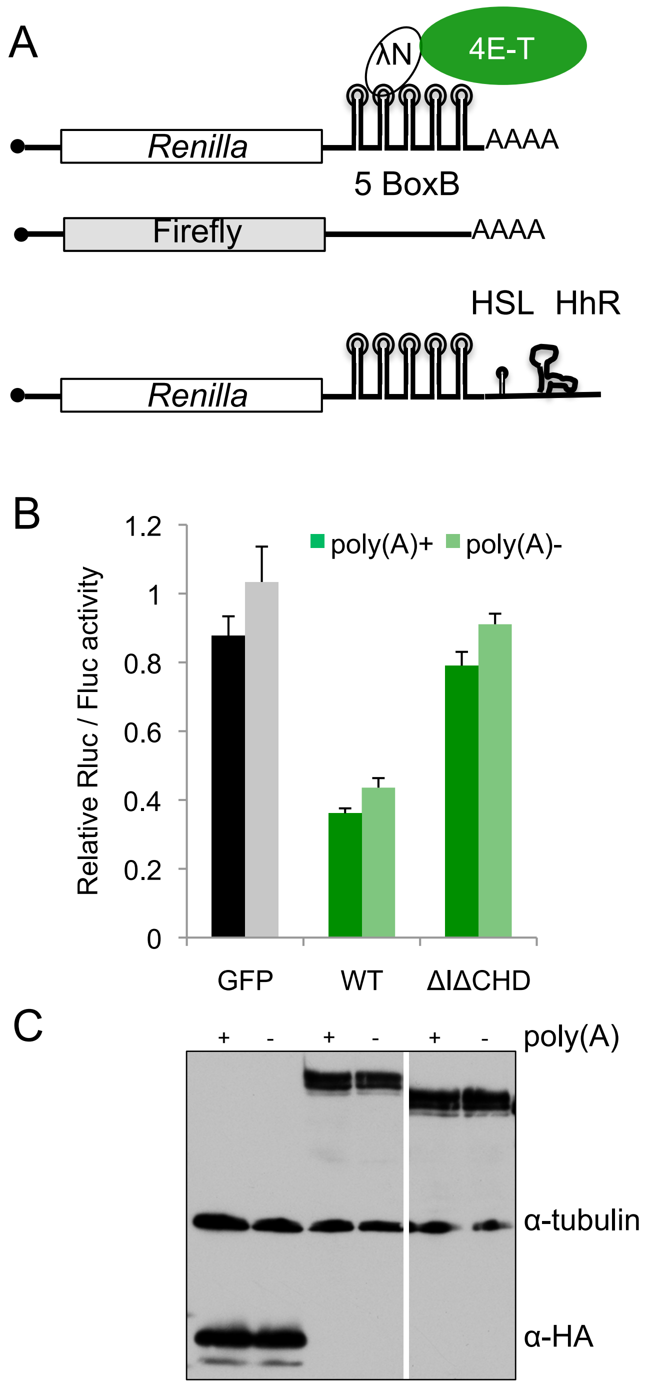
A
λN
4E-T
Renilla
AAAA
5 BoxB
Firefly
AAAA
HSL HhR
Renilla
B
Relative Rluc / Fluc activity
1.2
1
0.8
0.6
0.4
0.2
0
poly(A)+
poly(A)-
GFP
WT
ΔIΔCHD
C
+ - + - + -
poly(A)
α-tubulin
α-HA

Suppl. Fig. 4

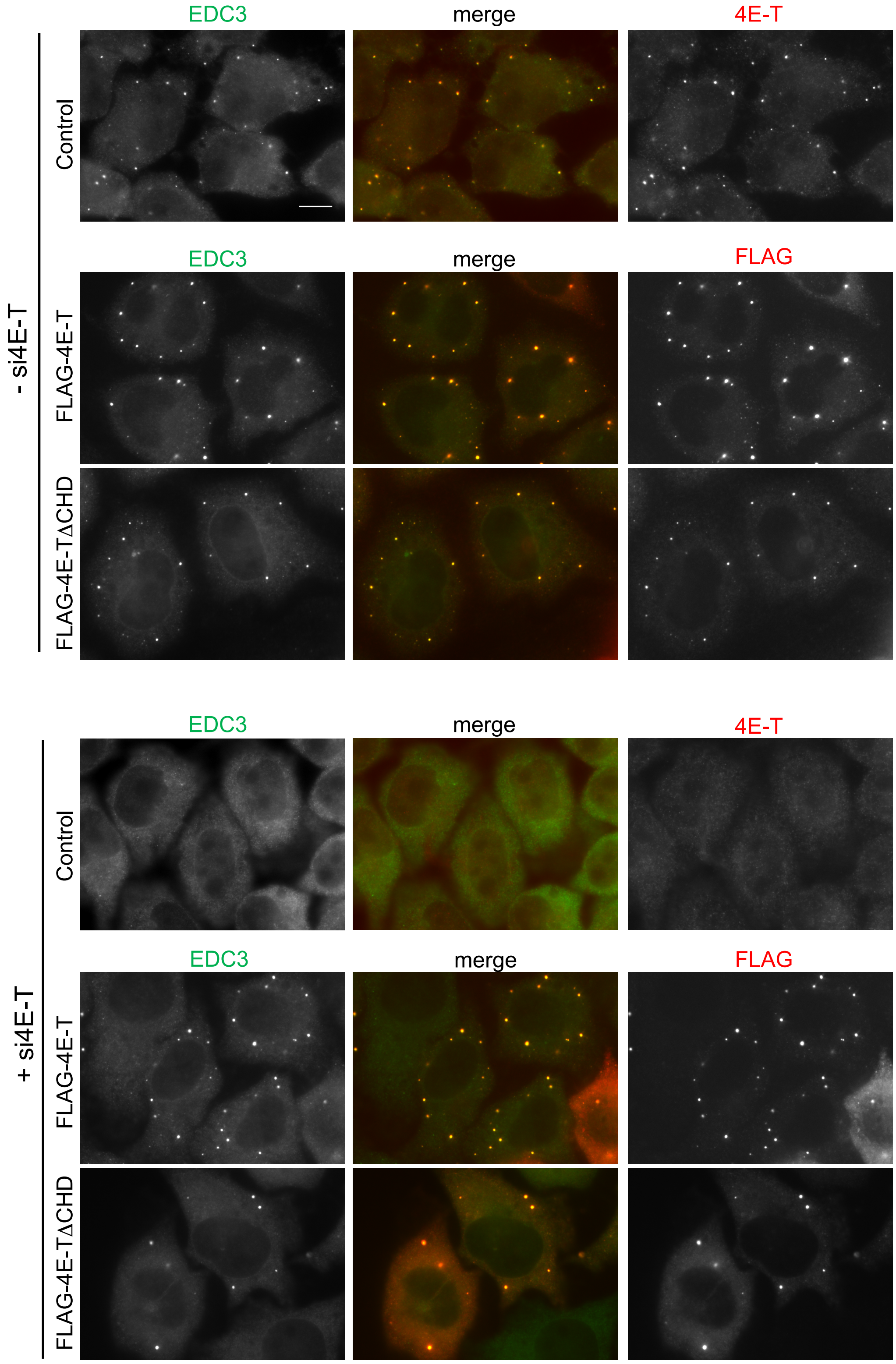
EDC3
merge
4E-T
FLAG
- si4E-T
+ si4E-T
Control
FLAG-4E-T
FLAG-4E-TΔCHD

Suppl. Fig. 5

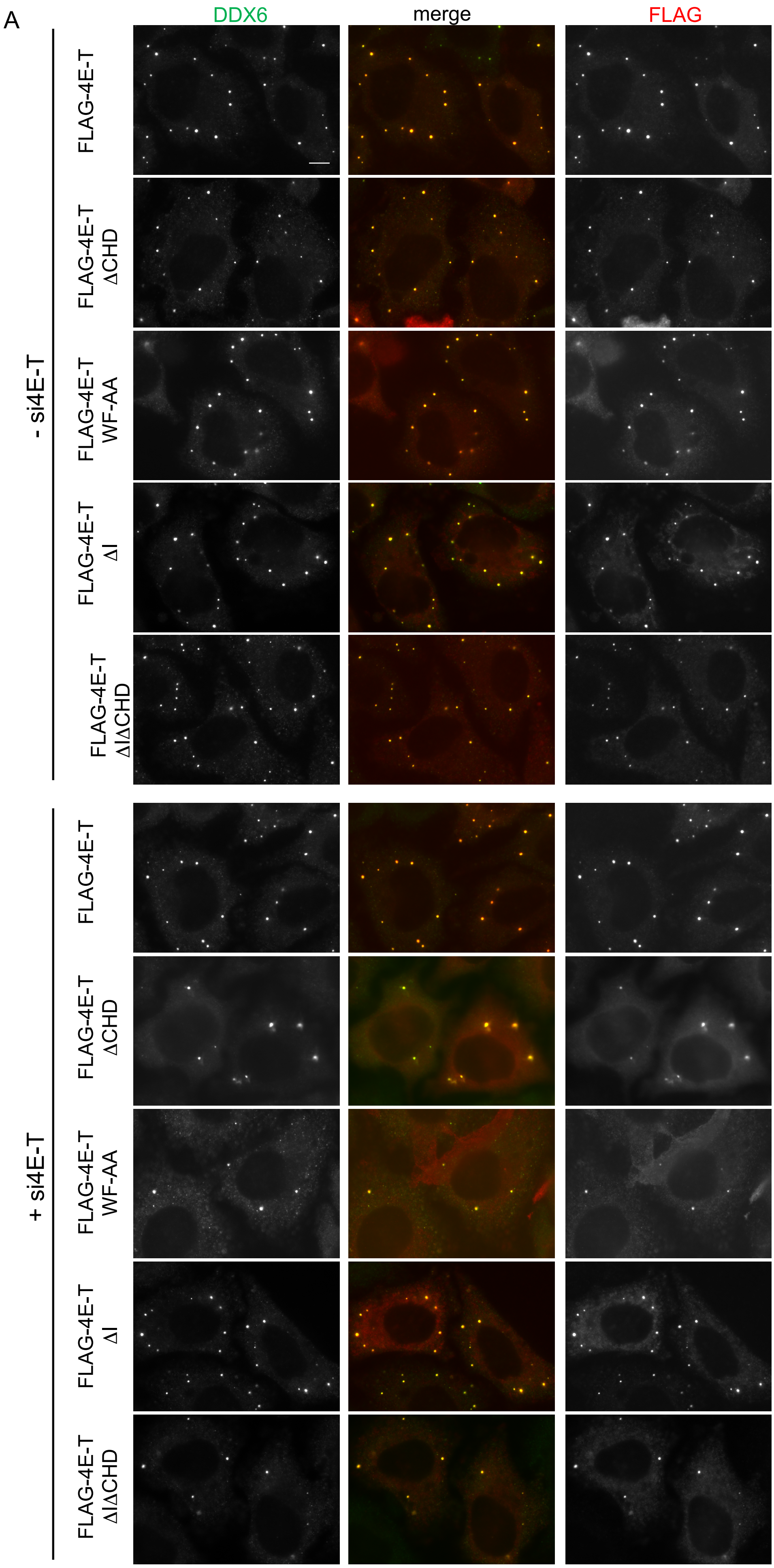
A
DDX6
merge
FLAG
- si4E-T
FLAG-4E-T
FLAG-4E-T ΔCHD
FLAG-4E-T WF-AA
FLAG-4E-T ΔI
FLAG-4E-T ΔIΔCHD
+ si4E-T
FLAG-4E-T
FLAG-4E-T ΔCHD
FLAG-4E-T WF-AA
FLAG-4E-T ΔI
FLAG-4E-T ΔIΔCHD

B

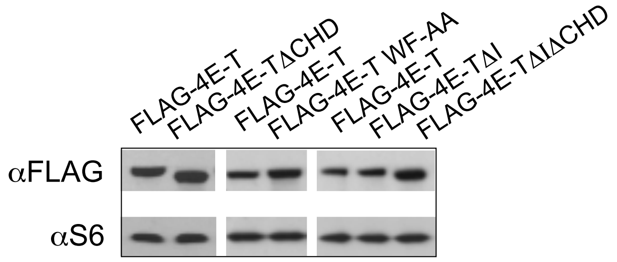
FLAG-4E-T
FLAG-4E-TΔCHD
FLAG-4E-T
FLAG-4E-T WF-AA
FLAG-4E-T
FLAG-4E-TΔI
FLAG-4E-TΔIΔCHD
αFLAG
αS6

Suppl. Fig. 6

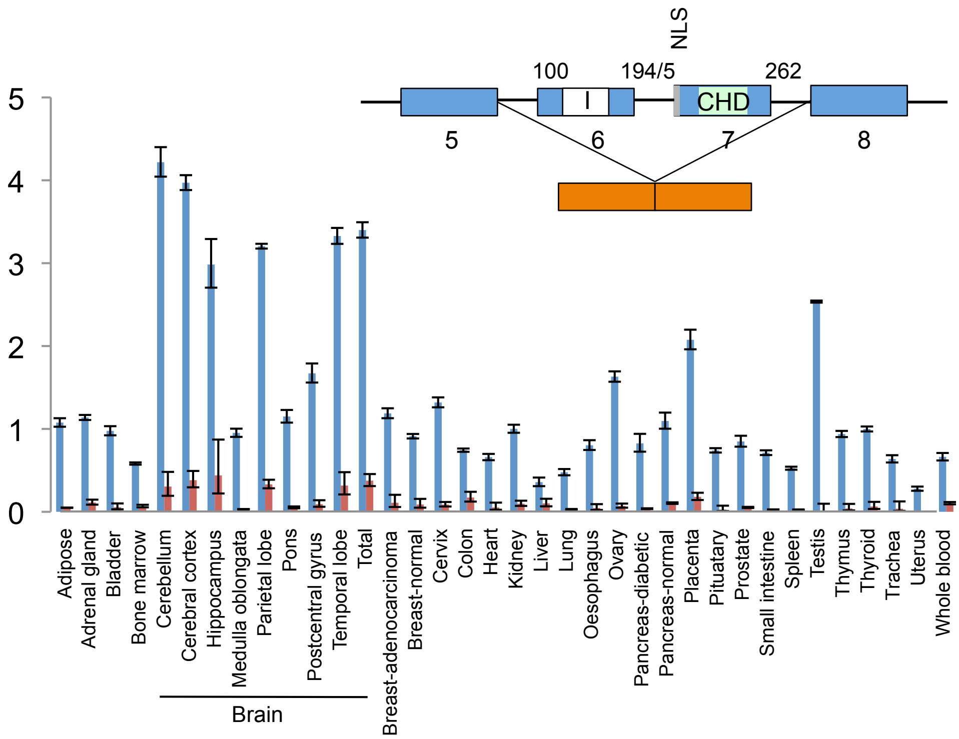
NLS
100
194/5
262
I
CHD
5
6
7
8
5
4
3
2
1
0
Adipose
Adrenal gland
Bladder
Bone marrow
Cerebellum
Cerebral cortex
Hippocampus
Medulla oblongata
Parietal lobe
Pons
Postcentral gyrus
Temporal lobe
Total
Breast-adenocarcinoma
Breast-normal
Cervix
Colon
Heart
Kidney
Liver
Lung
Oesophagus
Ovary
Pancreas-diabetic
Pancreas-normal
Placenta
Pituatary
Prostate
Small intestine
Spleen
Testis
Thymus
Thyroid
Trachea
Uterus
Whole blood
Brain

Suppl. Fig. 7

			Unique peptide sequences				sequence coverag					
Experiment			1	2	1	2	1	2	1	2	1	2
UNIPROT	GENE Official s	Description	4E-T	4E-T	Flag	Flag	4E-T	4E-T	PP 4E-T av	PP 4E-T av	EMPAI_CAL	EMPAI_CALC 4E-T avg
Q9NRA8	EIF4ENIF1	eukaryotic translation initiation factor 4E transporter isoform	58	72	5	0	68	66	17201	24369	5.61	6.4438
O75534	CSDE1	cold shock domain-containing protein E1 isoform 1	24	41	0	0	32	52	1953	3093	1.68	3.71
P26196	DDX6	probable ATP-dependent RNA helicase DDX6	9	31	0	0	28	71	688	2831	1.29	6.59
Q92598	HSPH1	heat shock protein 105 kDa isoform 1	20	30	0	0	31	45	1670	1731	1.49	2.83
Q9Y3F4	STRAP	serine-threonine kinase receptor-associated protein	20	29	1	0	78	67	2128	2778	6.08	6.94
P30153	PPP2R1A	serine/threonine-protein phosphatase 2A 65 kDa regulatory s	12	26	0	0	28	44	745	1924	1.48	3.98
P23396	RPS3	40S ribosomal protein S3 isoform 1	10	26	0	7	53	76	1124	1777	2.36	5.95
P06733	ENO1	alpha-enolase isoform 1	1	25	0	3	4	59	38	1255	0.10	4.25
P11940	PABPC1	polyadenylate-binding protein 1	9	25	1	13	18	39	395	1155	0.81	2.73
P31689	DNAJA1	dnaJ homolog subfamily A member 1	5	23	0	0	21	71	345	968	0.78	6.08
P49411	TUFM	Elongation factor Tu, mitochondrial	8	23	0	1	22	51	345	1256	0.81	3.10
Q5T9A4	ATAD3B	ATPase family AAA domain-containing protein 3B	6	22	0	0	11	38	337	833	0.40	2.07
P34932	HSPA4	heat shock 70 kDa protein 4	22	22	1	0	38	32	1669	1122	1.38	1.49
Q9UJS0	SLC25A13	calcium-binding mitochondrial carrier protein Aralar2 isoform	9	22	0	0	15	35	338	922	0.95	2.81
P05141	SLC25A5	ADP/ATP translocase 2	12	22	0	4	35	52	1221	2312	2.38	4.82
P35998	PSMC2	26S protease regulatory subunit 7 isoform 1	1	21	0	0	3	52	105	881	0.08	3.64
Q9Y230	RUVBL2	ruvB-like 2	9	21	0	0	23	55	519	1193	1.15	3.64
Q07065	CKAP4	cytoskeleton-associated protein 4	5	20	0	0	11	42	187	1322	0.32	1.83
P49368	CCT3	T-complex protein 1 subunit gamma isoform a	2	19	0	1	4	41	185	812	0.15	2.76
Q9Y262	EIF3L	eukaryotic translation initiation factor 3 subunit L isoform 1	8	19	0	0	19	36	440	900	0.78	2.65
P63151	PPP2R2A	serine/threonine-protein phosphatase 2A 55 kDa regulatory s	7	19	0	0	23	57	557	1447	0.78	3.22
Q06830	PRDX1	peroxiredoxin-1	9	19	2	3	47	79	501	1294	3.64	9.00
P13489	RNH1	ribonuclease inhibitor	6	19	0	0	19	56	481	779	0.67	4.05
P62701	RPS4X	40S ribosomal protein S4, X isoform X isoform	4	19	0	2	18	64	239	942	0.62	5.16
P12236	SLC25A6	ADP/ATP translocase 3	10	19	1	4	34	51	1106	1816	1.98	3.83
Q9BWD1	ACAT2	acetyl-CoA acetyltransferase, cytosolic	9	18	0	0	47	53	1012	1329	1.58	2.87
P61247	RPS3A	40S ribosomal protein S3a isoform 1	3	18	0	1	14	61	154	789	0.47	5.81
P08670	VIM	vimentin	1	18	0	0	2	42	45	785	0.07	2.27
P27348	YWHAQ	14-3-3 protein theta	8	18	0	0	38	53	683	1018	1.93	4.41
Q92841	DDX17	probable ATP-dependent RNA helicase DDX17 isoform 1	2	17	0	6	3	27	122	761	0.15	2.16
P68104	EEF1A1	elongation factor 1-alpha 1	8	17	1	4	24	37	526	1125	1.58	3.44
P60228	EIF3E	eukaryotic translation initiation factor 3 subunit E	19	17	0	0	48	41	1175	869	2.98	2.16
P36776	LONP1	lon protease homolog, mitochondrial isoform 1 precursor	10	17	0	0	16	24	611	542	0.64	1.40
P67775	PPP2CA	serine/threonine-protein phosphatase 2A catalytic subunit al	9	17	0	0	39	66	1013	1522	2.38	5.66
P62714	PPP2CB	serine/threonine-protein phosphatase 2A catalytic subunit be	9	17	1	0	39	66	895	1505	2.38	5.66
Q99873	PRMT1	Protein arginine N-methyltransferase 1	11	17	2	3	37	54	596	1090	2.36	5.16
O95831	AIFM1	apoptosis-inducing factor 1, mitochondrial isoform 1 precurs	7	16	0	0	16	33	400	769	0.74	2.56
Q99832	CCT7	T-complex protein 1 subunit eta isoform a	2	16	0	0	4	32	58	635	0.15	1.85
P13639	EEF2	elongation factor 2	4	16	0	0	6	23	236	629	0.23	1.27
P54652	HSPA2	heat shock-related 70 kDa protein 2	11	16	0	0	20	23	1323	2985	0.67	1.02
O95757	HSPA4L	heat shock 70 kDa protein 4L	10	16	0	0	17	27	605	699	0.49	0.89
P60891	PRPS1	ribose-phosphate pyrophosphokinase 1 isoform 1	3	16	0	0	11	54	92	855	0.54	4.62

Q00325	SLC25A3	phosphate carrier protein, mitochondrial isoform a precursor	6	16	0	0	19	34	355	644	1.00	2.55
P28288	ABCD3	ATP-binding cassette sub-family D member 3 isoform a	2	15	0	0	6	21	66	604	0.13	1.20
P11802	CDK4	cyclin-dependent kinase 4	2	15	0	0	9	55	55	1142	0.39	6.20
Q16531	DDB1	DNA damage-binding protein 1	10	15	0	0	11	17	398	482	0.49	0.86
Q13347	EIF3I	eukaryotic translation initiation factor 3 subunit I	14	15	1	0	64	59	689	711	3.64	4.28
Q15233	NONO	non-POU domain-containing octamer-binding protein isoforn	7	15	1	4	21	33	318	949	1.02	2.01
Q9P258	RCC2	protein RCC2	1	15	0	0	3	36	43	861	0.08	1.40
P39023	RPL3	60S ribosomal protein L3 isoform a	2	15	1	2	9	38	146	609	0.26	2.98
Q9Y265	RUVBL1	ruvB-like 1	4	15	0	0	11	42	210	630	0.55	3.64
P63104	YWHAZ	14-3-3 protein zeta/delta	5	15	0	1	26	47	402	652	1.05	3.87
Q53H12	AGK	acylglycerol kinase, mitochondrial precursor	8	14	0	0	27	36	382	615	1.68	2.73
P50991	CCT4	T-complex protein 1 subunit delta isoform a	3	14	0	1	8	30	128	548	0.24	1.55
P06493	CDK1	cyclin-dependent kinase 1 isoform 1	2	14	0	0	7	54	92	451	0.25	2.73
Q96EY1	DNAJA3	dnaJ homolog subfamily A member 3, mitochondrial isoform	7	14	0	0	18	28	312	617	1.08	1.57
Q99615	DNAJC7	dnaJ homolog subfamily C member 7 isoform 1	1	14	0	0	2	31	82	597	0.07	1.58
O15371	EIF3D	eukaryotic translation initiation factor 3 subunit D	7	14	0	2	21	33	386	724	1.31	2.90
P63244	GNB2L1	guanine nucleotide-binding protein subunit beta-2-like 1	1	14	0	1	5	57	56	753	0.27	4.46
P22234	PAICS	multifunctional protein ADE2 isoform 2	1	14	0	0	3	37	111	539	0.12	3.16
Q53H96	PYCRL	Pyrroline-5-carboxylate reductase 3	6	14	0	0	27	57	617	947	2.16	7.25
Q16576	RBBP7	histone-binding protein RBBP7 isoform 2	4	14	0	0	14	48	256	1135	0.58	4.41
Q9Y5M8	SRPRB	signal recognition particle receptor subunit beta	9	14	0	0	46	56	653	850	2.16	3.64
P40227	CCT6A	T-complex protein 1 subunit zeta isoform a	1	13	0	2	2	24	40	450	0.09	2.16
P60842	EIF4A1	eukaryotic initiation factor 4A-I isoform 1	3	13	0	0	10	43	236	698	0.35	2.67
P38919	EIF4A3	eukaryotic initiation factor 4A-III	5	13	1	1	15	29	225	362	0.62	1.87
P42704	LRPPRC	leucine-rich PPR motif-containing protein, mitochondrial prec	4	13	0	0	3	10	100	426	0.10	0.37
P62195	PSMC5	26S protease regulatory subunit 8 isoform 1	3	13	0	1	9	45	127	628	0.32	2.63
P62333	PSMC6	26S protease regulatory subunit 10B	1	13	0	0	4	42	94	515	0.11	2.01
P36578	RPL4	60S ribosomal protein L4	4	13	1	1	11	32	129	497	0.55	2.34
P62424	RPL7A	60S ribosomal protein L7a	5	13	0	2	22	41	240	439	1.42	3.92
P15880	RPS2	40S ribosomal protein S2	5	13	1	1	18	41	171	650	0.78	2.55
P31946	YWHAB	14-3-3 protein beta/alpha	4	13	0	0	17	47	308	547	0.85	3.64
P25705	ATP5A1	ATP synthase subunit alpha, mitochondrial isoform a precurs	8	12	0	4	20	24	519	700	0.78	1.21
Q9Y3I0	C22orf28,RTCB	tRNA-splicing ligase RtcB homolog	5	12	0	0	11	29	216	510	0.56	2.16
P48643	CCT5	T-complex protein 1 subunit epsilon	3	12	0	1	8	30	297	390	0.25	1.44
O15372	EIF3H	eukaryotic translation initiation factor 3 subunit H	5	12	0	0	17	45	450	590	0.73	2.73
P50402	EMD	emerin	9	12	0	0	39	58	577	579	3.64	4.62
P62993	GRB2	growth factor receptor-bound protein 2 isoform 1	5	12	0	0	26	52	245	862	1.42	4.88
O14929	HAT1	histone acetyltransferase type B catalytic subunit	10	12	0	0	30	33	810	1344	1.82	2.16
P30041	PRDX6	peroxiredoxin-6	1	12	0	1	4	60	61	516	0.17	3.64
Q00610	CLTC	clathrin heavy chain 1 isoform 1	4	11	0	0	3	10	132	430	0.12	0.37
P61962	DCAF7	DDB1- and CUL4-associated factor 7	4	11	0	0	16	36	403	686	0.85	4.41
Q9HAV7	GRPEL1	grpE protein homolog 1, mitochondrial precursor	6	11	0	0	34	51	475	807	1.37	2.65
Q9BX40	LSM14B	protein LSM14 homolog B	3	11	0	0	10	51	114	690	0.58	2.41
Q13310	PABPC4	polyadenylate-binding protein 4 isoform 2	0	11	0	5	0	19		472		0.84
Q9UNM6	PSMD13	26S proteasome non-ATPase regulatory subunit 13 isoform 1	3	11	0	0	8	31	120	418	0.35	2.01

Q9UHX1	PUF60	poly(U)-binding-splicing factor PUF60 isoform a	9	11	0	1	22	29	448	514	1.15	1.87
P18124	RPL7	60S ribosomal protein L7	5	11	2	1	20	44	267	406	1.05	3.22
P62753	RPS6	40S ribosomal protein S6	3	11	1	2	14	34	214	642	0.87	2.51
P46781	RPS9	40S ribosomal protein S9	6	11	0	0	26	41	200	412	1.68	2.73
Q9UBX3	SLC25A10	mitochondrial dicarboxylate carrier isoform 2	8	11	0	0	29	39	396	597	2.16	3.87
P06753	TPM3	tropomyosin alpha-3 chain isoform 1	1	11	0	1	5	31	59	339	0.10	1.15
Q9BUF5	TUBB6	tubulin beta-6 chain	4	11	0	0	11	28	314	717	0.62	1.64
P61981	YWHAG	14-3-3 protein gamma	5	11	0	1	21	39	299	391	1.05	3.22
Q9NRN7	AASDHPPT	L-aminoadipate-semialdehyde dehydrogenase-phosphopante	3	10	0	0	13	35	141	335	0.54	3.22
P52907	CAPZA1	F-actin-capping protein subunit alpha-1	2	10	0	0	9	55	67	467	0.36	2.41
Q9H3G5	CPVL	probable serine carboxypeptidase CPVL precursor	4	10	0	0	9	22	460	624	0.49	1.46
O00303	EIF3F	eukaryotic translation initiation factor 3 subunit F	4	10	0	2	15	34	257	509	1.03	4.88
P06730	EIF4E	eukaryotic translation initiation factor 4E isoform 1	5	10	0	0	27	37	508	735	2.16	4.01
P52597	HNRNPF	heterogeneous nuclear ribonucleoprotein F	4	10	0	2	14	40	261	652	0.72	2.38
P28482	MAPK1	mitogen-activated protein kinase 1	4	10	0	0	13	32	95	428	0.55	1.99
P22061	PCMT1	Protein-L-isoaspartate(D-aspartate) O-methyltransferase	1	10	0	0	4	62	65	521	0.26	5.31
Q13162	PRDX4	peroxiredoxin-4 precursor	7	10	0	0	37	48	337	776	2.46	3.92
O14818	PSMA7	proteasome subunit alpha type-7	3	10	2	0	21	51	256	393	0.64	3.39
Q9P035	PTPLAD1	very-long-chain (3R)-3-hydroxyacyl-[acyl-carrier protein] dehy	5	10	0	0	17	26	255	403	1.28	2.73
P04843	RPN1	dolichyl-diphosphooligosaccharide--protein glycosyltransfera	3	10	0	3	6	19	117	474	0.22	0.93
P62249	RPS16	40S ribosomal protein S16	1	10	0	1	7	49	34	600	0.26	4.01
P46782	RPS5	40S ribosomal protein S5	3	10	0	0	22	28	346	641	1.15	2.59
Q9UNE7	STUB1	E3 ubiquitin-protein ligase CHIP isoform a	2	10	0	0	7	38	111	308	0.25	2.34
Q9H7D7	WDR26	WD repeat-containing protein 26 isoform a	1	10	0	0	2	18	101	412	0.07	1.05
P24539	ATP5F1	ATP synthase F(0) complex subunit B1, mitochondrial precurs	3	9	0	0	14	33	239	512	0.54	1.74
P48047	ATP5O	ATP synthase subunit O, mitochondrial precursor	5	9	0	0	27	54	301	471	1.42	3.12
Q9Y224	C14orf166	UPF0568 protein C14orf166	8	9	0	0	44	44	720	377	1.78	2.59
O75821	EIF3G	eukaryotic translation initiation factor 3 subunit G	3	9	0	0	9	29	212	342	0.31	1.25
Q7L2H7	EIF3M	eukaryotic translation initiation factor 3 subunit M	5	9	0	0	21	31	338	583	0.62	1.98
Q99714	HSD17B10	3-hydroxyacyl-CoA dehydrogenase type-2 isoform 1	5	9	0	0	33	48	177	518	1.42	3.12
Q96CX2	KCTD12	BTB/POZ domain-containing protein KCTD12	1	9	0	0	4	28	45	304	0.15	1.74
Q14739	LBR	lamin-B receptor	1	9	0	0	3	16	83	248	0.12	1.15
Q96AG4	LRRC59	leucine-rich repeat-containing protein 59	7	9	0	0	30	35	411	445	1.58	1.96
P35232	PHB	prohibitin isoform 1	6	9	0	3	31	34	500	341	1.25	2.38
P32119	PRDX2	peroxiredoxin-2	5	9	0	0	24	34	319	472	1.51	2.98
P25789	PSMA4	proteasome subunit alpha type-4 isoform 1	3	9	0	2	10	38	170	451	0.70	3.12
P31040	SDHA	succinate dehydrogenase [ubiquinone] flavoprotein subunit,	3	9	0	0	6	21	172	454	0.27	0.89
Q96BR5	SELRC1,COA7	cytochrome c oxidase assembly factor 7	3	9	0	0	16	45	101	330	0.50	1.96
P31930	UQCRC1	cytochrome b-c1 complex subunit 1, mitochondrial precursor	2	9	0	0	5	23	105	395	0.23	1.31
P45880	VDAC2	voltage-dependent anion-selective channel protein 2 isoform	6	9	0	3	25	38	285	633	1.51	1.93
Q04917	YWHAH	14-3-3 protein eta	4	9	0	0	16	39	257	307	0.78	3.22
P35613	BSG	basigin isoform 1 precursor	5	8	0	0	15	26	218	454	1.03	2.46
Q6IAN0	DHRS7B	dehydrogenase/reductase SDR family member 7B	2	8	0	0	8	28	221	379	0.31	1.96
Q9NWT6	HIF1AN	hypoxia-inducible factor 1-alpha inhibitor	6	8	0	0	27	21	433	337	1.37	1.74
P25786	PSMA1	proteasome subunit alpha type-1 isoform 2	1	8	0	0	10	37	38	276		1.96

O00231	PSMD11	26S proteasome non-ATPase regulatory subunit 11	2	8	0	1	5	20	64	293	0.19	0.98
O00232	PSMD12	26S proteasome non-ATPase regulatory subunit 12 isoform 1	1	8	0	0	3	18	97	321	0.08	0.74
P62913	RPL11	60S ribosomal protein L11 isoform 1	2	8	0	2	13	35	110	363	0.67	2.59
Q8NC51	SERBP1	plasminogen activator inhibitor 1 RNA-binding protein isoforı	1	8	0	0	4	22	73	306	0.12	1.24
Q96J01	THOC3	THO complex subunit 3	1	8	0	0	3	24	50	297	0.15	1.96
P49755	TMED10	transmembrane emp24 domain-containing protein 10 precur	1	8	0	0	5	37	61	400	0.23	1.85
P36542	ATP5C1	ATP synthase subunit gamma, mitochondrial isoform L (liver)	5	7	0	1	19	23	268	581	1.42	2.46
O95816	BAG2	BAG family molecular chaperone regulator 2	3	7	0	0	15	32	176	442	0.54	1.74
P47756	CAPZB	F-actin-capping protein subunit beta isoform 2	1	7	0	1	5	29	58	489	0.14	1.45
Q9BT78	COPS4	COP9 signalosome complex subunit 4 isoform 1	2	7	0	0	5	20	85	345	0.18	0.78
Q13838	DDX39B	spliceosome RNA helicase DDX39B	4	7	0	1	8	20	125	264	0.45	0.91
O60884	DNAJA2	dnaJ homolog subfamily A member 2	2	7	0	0	9	15	144	422	0.27	1.07
O60573	EIF4E2	eukaryotic translation initiation factor 4E type 2 isoform A	3	7	0	0	20	34	134	268	0.87	2.51
Q12905	ILF2	interleukin enhancer-binding factor 2 isoform 1	2	7	0	0	9	25	111	269	0.36	1.93
P00338	LDHA	L-lactate dehydrogenase A chain isoform 1	3	7	0	0	11	21	153	342	0.37	1.08
Q15365	PCBP1	poly(rC)-binding protein 1	4	7	0	0	14	26	233	482	0.78	1.74
Q9BRX2	PELO	protein pelota homolog	2	7	0	0	8	21	52	215	0.23	1.08
O43175	PHGDH	D-3-phosphoglycerate dehydrogenase	3	7	0	0	6	18	162	346	0.39	1.15
P25788	PSMA3	proteasome subunit alpha type-3 isoform 1	2	7	0	0	8	29	149	271	0.39	2.16
P28066	PSMA5	proteasome subunit alpha type-5 isoform 1	4	7	0	0	17	37	251	307	1.31	3.33
P28074	PSMB5	proteasome subunit beta type-5 isoform 1	2	7	1	0	9	34	77	547	0.36	1.93
Q9NXS2	QPCTL	glutaminyl-peptide cyclotransferase-like protein isoform 1	2	7	0	0	5	15	124	283	0.43	1.42
P51149	RAB7A	ras-related protein Rab-7a	7	7	0	1	40	50	181	251	1.58	1.96
Q9NZ01	TECR	very-long-chain enoyl-CoA reductase	3	7	0	0	9	17	182	275	0.54	1.05
P26368	U2AF2	splicing factor U2AF 65 kDa subunit isoform a	6	7	0	1	19	25	307	334	1.37	1.37
Q9UL15	BAG5	BAG family molecular chaperone regulator 5 isoform b	3	6	0	0	11	16	116	249	0.30	0.70
Q13185	C15orf57,CBX3	chromobox protein homolog 3	2	6	0	0	16	38	102	372	0.78	3.22
Q9BSF4	C19orf52	uncharacterized protein C19orf52	5	6	0	0	30	28	480	225	1.42	1.89
Q07021	C1QBP	complement component 1 Q subcomponent-binding protein,	7	6	1	1	41	33	597	506	1.31	1.31
Q92499	DDX1	ATP-dependent RNA helicase DDX1	2	6	0	0	5	10	76	213	0.11	0.37
O75190	DNAJB6	dnaJ homolog subfamily B member 6 isoform a	5	6	0	0	18	23	301	391	1.28	1.68
P29692	EEF1D	elongation factor 1-delta isoform 2	2	6	0	2	9	30	116	275	0.29	1.15
Q9NZL4	HSPBP1	Hsp70-binding protein 1	5	6	0	0	17	22	198	260	1.05	1.37
Q01081	LOC10272459	splicing factor U2AF 35 kDa subunit isoform a	4	6	0	0	20	34	215	215	1.31	2.51
Q9Y383	LUC7L2	putative RNA-binding protein Luc7-like 2 isoform 1	6	6	0	0	18	16	497	258	1.15	0.90
Q15366	PCBP2	poly(rC)-binding protein 2 isoform d	4	6	0	0	15	23	218	446	0.85	1.51
Q96HS1	PGAM5	serine/threonine-protein phosphatase PGAM5, mitochondria	3	6	0	0	11	22	121	262	0.47	1.15
Q9Y2S7	POLDIP2	polymerase delta-interacting protein 2 isoform 1	2	6	0	0	6	20	52	226	0.25	0.93
P30048	PRDX3	thioredoxin-dependent peroxide reductase, mitochondrial isc	4	6	0	0	18	24	244	355	1.31	2.51
P60900	PSMA6	proteasome subunit alpha type-6 isoform a	2	6	1	0	9	28	140	337	0.47	2.16
P62491	RAB11A	ras-related protein Rab-11A isoform 1	4	6	0	0	20	28	224	361	1.03	1.89
P51148	RAB5C	ras-related protein Rab-5C isoform a	4	6	0	0	22	43	231	365	1.51	2.98
P61313	RPL15	60S ribosomal protein L15 isoform 1	2	6	1	0	10	29	107	235	0.43	1.89
P18621	RPL17	60S ribosomal protein L17 isoform a	2	6	0	1	14	30	110	420	0.52	1.85
P83731	RPL24	60S ribosomal protein L24	2	6	1	1	13	37	157	317	0.78	3.22

P61619	SEC61A1	protein transport protein Sec61 subunit alpha isoform 1	3	6	0	0	6	11	68	213	0.64	1.28
P09661	SNRPA1	U2 small nuclear ribonucleoprotein A'	4	6	1	1	18	22	128	176	1.31	1.85
P57088	TMEM33	transmembrane protein 33	4	6	0	0	16	23	203	383	1.78	3.64
Q9H3P7	ACBD3	Golgi resident protein GCP60	2	5	0	0	8	16	135	234	0.27	1.07
O75947	ATP5H	ATP synthase subunit d, mitochondrial isoform a	6	5	0	0	40	22	263	170	2.98	1.51
P54105	CLNS1A	methylosome subunit pICln	2	5	1	6	19	43	107	466	1.00	4.01
P49366	DHPS	deoxyhypusine synthase isoform a	2	5	0	0	6	18	97	145	0.23	0.69
Q96F86	EDC3	enhancer of mRNA-decapping protein 3	0	5	0	0	0	13		177		0.51
Q99613	EIF3C	eukaryotic translation initiation factor 3 subunit C isoform a	6	5	0	0	7	7	236	209	0.41	0.33
Q92552	MRPS27	28S ribosomal protein S27, mitochondrial isoform 2	4	5	0	0	13	15	146	137	0.45	0.74
O75489	NDUFS3	NADH dehydrogenase [ubiquinone] iron-sulfur protein 3, mit	4	5	0	0	20	22	203	249	0.72	0.97
Q86TB9	PATL1	protein PAT1 homolog 1	1	5	0	0	1	7	80	216	0.07	0.42
O00264	PGRMC1	membrane-associated progesterone receptor component 1 is	4	5	0	0	37	21	288	297	1.15	1.78
Q9H0U4	RAB1B	ras-related protein Rab-1B	5	5	0	0	34	34	236	227	1.15	1.15
P84098	RPL19	60S ribosomal protein L19	2	5	1	0	13	18	140	283	1.15	2.16
O75396	SEC22B	Vesicle-trafficking protein SEC22b	2	5	0	0	9	26	75	332	0.43	1.42
P53007	SLC25A1	tricarboxylate transport protein, mitochondrial isoform a pre	3	5	0	0	12	17	102	172	0.54	1.05
Q02978	SLC25A11	mitochondrial 2-oxoglutarate/malate carrier protein isoform	4	5	0	0	16	16	215	212	0.67	0.90
Q9Y4P3	TBL2	transducin beta-like protein 2 precursor	3	5	0	0	8	14	134	232	0.29	0.53
Q3ZCQ8	TIMM50	Mitochondrial import inner membrane translocase subunit T	5	5	0	0	18	14	191	270	1.42	1.03
Q9Y277	VDAC3	voltage-dependent anion-selective channel protein 3 isoform	2	5	0	0	8	22	133	265	0.36	1.15
O95573	ACSL3	long-chain-fatty-acid--CoA ligase 3	3	4	0	0	6	7	77	177	0.23	0.32
P55884	EIF3B	eukaryotic translation initiation factor 3 subunit B	5	4	0	0	9	6	191	133	0.39	0.30
Q9UBQ5	EIF3K	eukaryotic translation initiation factor 3 subunit K	3	4	0	1	19	24	288	262	0.87	0.87
Q15717	ELAVL1	ELAV-like protein 1	2	4	0	1	7	14	121	244	0.33	0.78
P13995	MTHFD2	bifunctional methylenetetrahydrofolate dehydrogenase/cyclo	2	4	0	0	10	24	101	135	0.27	0.62
O43809	NUDT21	cleavage and polyadenylation specificity factor subunit 5	3	4	0	0	15	21	106	119	0.64	0.93
O75439	PMPCB	mitochondrial-processing peptidase subunit beta precursor	2	4	0	0	4	10	71	195	0.19	0.43
P61026	RAB10	ras-related protein Rab-10	2	4	0	0	11	18	142	186	0.36	0.85
Q92600	RQCD1	cell differentiation protein RCD1 homolog isoform 2	2	4	0	0	8	14	154	162	0.27	0.62
P84103	SRSF3	serine/arginine-rich splicing factor 3	2	4	0	0	14	29	135	119	0.67	1.78
Q6DKK2	TTC19	tetratricopeptide repeat protein 19, mitochondrial isoform 1	6	4	0	0	16	10	287	112	0.87	0.52
P0CG48	UBC	polyubiquitin-C	2	4	0	0	33	53	254	509	0.93	2.73
Q9UBS4	DNAJB11	dnaJ homolog subfamily B member 11 precursor	2	3	0	0	8	7	114	190	0.31	0.31
P55084	HADHB	trifunctional enzyme subunit beta, mitochondrial isoform 1 p	4	3	0	0	10	7	181	140	0.47	0.33
Q8ND56	LSM14A	protein LSM14 homolog A isoform a	2	3	0	0	6	9	53	118	0.29	0.47
O75306	NDUFS2	NADH dehydrogenase [ubiquinone] iron-sulfur protein 2, mit	2	3	0	0	4	8	86	116	0.21	0.33
Q8NFH4	NUP37	nucleoporin Nup37	2	3	0	0	10	12	118	115	0.43	0.70
P19387	POLR2C	DNA-directed RNA polymerase II subunit RPB3	2	3	0	0	7	14	109	107	0.39	0.64
P61106	RAB14	ras-related protein Rab-14	2	3	0	0	11	16	125	174	0.39	0.64
P53985	SLC16A1	monocarboxylate transporter 1	3	3	0	0	11	7	92	79	1.37	0.78
Q9UJZ1	STOML2	stomatin-like protein 2, mitochondrial isoform a	3	3	0	0	13	12	152	162	0.54	0.54
O95292	VAPB	vesicle-associated membrane protein-associated protein B/C	2	3	0	0	12	15	85	137	0.36	0.58
P51572	BCAP31	B-cell receptor-associated protein 31 isoform b	4	2	0	0	13	10	163	82	0.70	0.70
P00403	COX2	Cytochrome c oxidase subunit 2	2	2	0	0	11	7	78	76	1.15	1.15

Q9UBM7	DHCR7	7-dehydrocholesterol reductase	2	2	0	0	4	4	69	63	0.43	0.43
Q9NXW2	DNAJB12	DnaJ homolog subfamily B member 12	3	2	0	0	10	7	137	86	0.47	0.29
Q14152	EIF3A	eukaryotic translation initiation factor 3 subunit A	9	2	0	0	8	1	546	57	0.27	0.05
O94905	ERLIN2	erlin-2 isoform 1	3	2	0	0	12	7	193	119	0.39	0.25
P31942	HNRNPH3	heterogeneous nuclear ribonucleoprotein H3 isoform a	2	2	0	0	9	6	97	95	0.27	0.27
O14654	IRS4	insulin receptor substrate 4	2	2	0	0	2	1	66	116	0.09	0.09
Q9H7Z7	PTGES2	prostaglandin E synthase 2 isoform 1	2	2	0	0	6	6	69	99	0.21	0.21
Q96I25	RBM17	splicing factor 45	2	2	0	0	5	4	63	68	0.25	0.25
Q96E39	RBMXL1	RNA binding motif protein, X-linked-like-1	2	2	0	0	6	7	49	88	0.18	0.18
P21912	SDHB	succinate dehydrogenase [ubiquinone] iron-sulfur subunit, m	3	2	0	0	11	8	76	124	0.44	0.27
Q15363	TMED2	transmembrane emp24 domain-containing protein 2 precurs	2	2	0	0	11	9	224	134	0.67	0.67
Q9BVK6	TMED9	transmembrane emp24 domain-containing protein 9 precurs	3	2	0	0	15	9	205	125	0.54	0.33
